# Integrated Environmental and Genomic Analysis Reveals the Drivers of Local Adaptation in African Indigenous Chickens

**DOI:** 10.1093/molbev/msab156

**Published:** 2021-05-22

**Authors:** Almas A Gheyas, Adriana Vallejo-Trujillo, Adebabay Kebede, Maria Lozano-Jaramillo, Tadelle Dessie, Jacqueline Smith, Olivier Hanotte

**Affiliations:** 1 Centre for Tropical Livestock Genetics and Health (CTLGH), The Roslin Institute, University of Edinburgh, Edinburgh, United Kingdom; 2 Cells, Organism and Molecular Genetics, School of Life Sciences, University of Nottingham, Nottingham, United Kingdom; 3 LiveGene—CTLGH, International Livestock Research Institute (ILRI) Ethiopia, Addis Ababa, Ethiopia; 4 Amhara Regional Agricultural Research Institute, Bahir Dar, Ethiopia; 5 Wageningen University & Research Animal Breeding and Genomics, Wageningen, The Netherlands

**Keywords:** local environmental adaptation, ecological niche modeling, selection signature, genotype−environment association, redundancy analysis, African indigenous chicken

## Abstract

Breeding for climate resilience is currently an important goal for sustainable livestock production. Local adaptations exhibited by indigenous livestock allow investigating the genetic control of this resilience. Ecological niche modeling (ENM) provides a powerful avenue to identify the main environmental drivers of selection. Here, we applied an integrative approach combining ENM with genome-wide selection signature analyses (*XPEHH* and *Fst*) and genotype−environment association (redundancy analysis), with the aim of identifying the genomic signatures of adaptation in African village chickens. By dissecting 34 agro-climatic variables from the ecosystems of 25 Ethiopian village chicken populations, ENM identified six key drivers of environmental challenges: One temperature variable—strongly correlated with elevation, three precipitation variables as proxies for water availability, and two soil/land cover variables as proxies of food availability for foraging chickens. Genome analyses based on whole-genome sequencing (*n* = 245), identified a few strongly supported genomic regions under selection for environmental challenges related to altitude, temperature, water scarcity, and food availability. These regions harbor several gene clusters including regulatory genes, suggesting a predominantly oligogenic control of environmental adaptation. Few candidate genes detected in relation to heat-stress, indicates likely epigenetic regulation of thermo-tolerance for a domestic species originating from a tropical Asian wild ancestor. These results provide possible explanations for the rapid past adaptation of chickens to diverse African agro-ecologies, while also representing new landmarks for sustainable breeding improvement for climate resilience. We show that the pre-identification of key environmental drivers, followed by genomic investigation, provides a powerful new approach for elucidating adaptation in domestic animals.

## Background

The global livestock sector is facing a major threat from climate change. Extreme weather and global warming are not only challenging the physiological tolerance of animals but also adversely affecting their ecosystems, leading to changes in the quality and quantity of livestock feed or forage, water availability, and disease prevalence (Rojas-Downing et al. 2017; Rashamol and Sejian 2018). The demand for livestock products, however, is on rise and is estimated to double by 2050 due to increasing populations and improved living standards (Rashamol and Sejian 2018). Sustainable improvement of livestock production, to cater for the increased global demand, will therefore crucially depend on our ability to utilize/develop climate resilient breeds. Indigenous livestock populations in different parts of the world show greater adaptation to their local agro-climatic conditions compared with exotic breeds (Rashamol and Sejian 2018). Some livestock species, like domestic chicken, show wide environmental tolerance as they are found in practically all human settlements around the world—both in tropical and temperate regions. Elucidating the genetic components of local adaptations in such ubiquitous species will be invaluable toward achieving climate change resilience by allowing the identification of stress adaptation genes and thereby facilitating breed improvements by combining productivity and resilience genotypes.

**Fig. 1.**
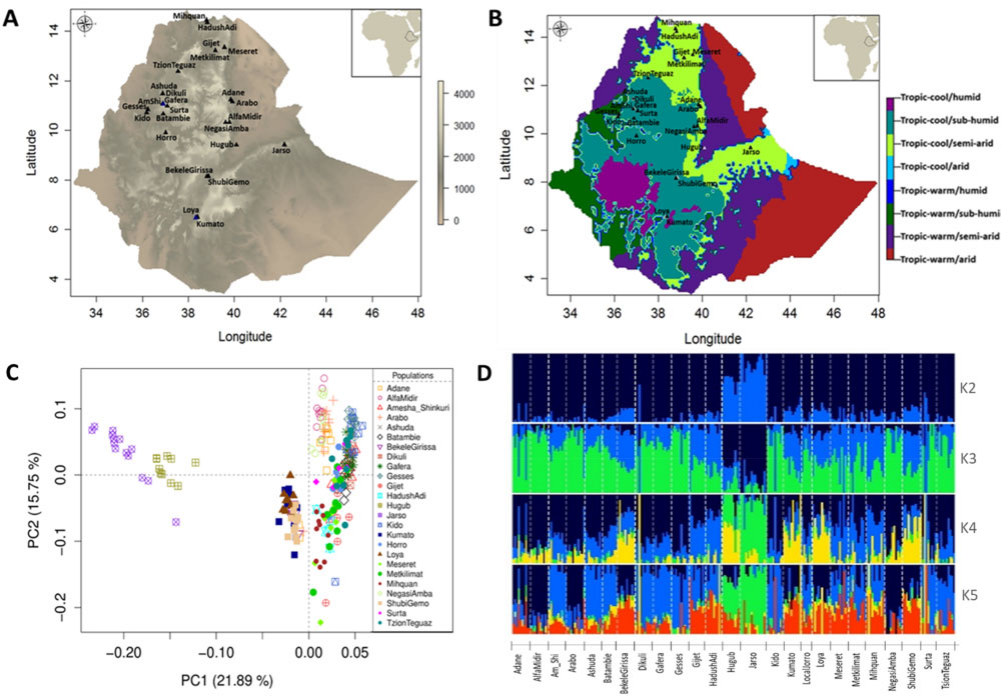
(*A*, *B*) Sampling location of Ethiopian indigenous chicken populations in relation to variation in elevation and agro-ecological zones—AEZ ( HarvestChoice 2015); (*C*) PCA plots of the populations based on 14 million autosomal SNPs; (*D*) admixture analysis results for *K* values between 2 and 5 (best *K *=* *3).

In the present study, we dissect the environmental and genomic data of many Ethiopian indigenous chickens from diverse agro-climatic regions to identify the environmental and genetic drivers of local adaptation. Ethiopia, with its extreme altitudinal topographies—varying from below sea level to over 4,500 m above sea level (m.a.s.l)—illustrates the diversity of agro-ecologies found across tropical Africa (fig. 1*A* and *B*). Three distinct temperature zones are observed in Ethiopia—cool (dega), temperate (weina dega), and hot (kolla) (The Library of Congress 1991). The cool zone expands over the western and the eastern parts of the north-western Ethiopian plateau with elevation generally above 2,400 m.a.s.l., and temperature between near-freezing and 16 °C. Lower elevations of the plateau (1,500−2,400 m.a.s.l.) constitute the temperate zone, where temperature varies between 16 °C and 30 °C. The hot zone is located mostly in the eastern parts of the country, where the elevation is below 1,500 m.a.s.l., and the maximum temperature can reach as high as 50 °C. A large variation in precipitation—from about 15 to 210 cm per annum—is also observed across the country (Fazzini et al. 2015). Although some areas receive rainfall throughout the year, in other parts it is mostly seasonal. Rainfall is the heaviest and most abundant in the southwest and generally decreases from South to North, mainly along the eastern lowlands (The Library of Congress 1991). Different combinations of temperature and rainfall patterns have created a gradation in climatic conditions, which vary from hot-humid and hot-arid to cold-humid and cold-arid (fig. 1*B*).

The chicken (*Gallus gallus domesticus)* is an introduced species in Africa. Although Egypt provides the earliest iconographic evidence of domestic chicken (Mwacharo et al. 2013), the oldest African chicken bones were found in Ethiopia at the Mezber site of Tigray region, dated to around c.921–801 BCE (Woldekiros and D'Andrea 2017). Molecular evidence supports at least two arrival/dispersion waves for domestic chickens in Africa (Mwacharo et al. 2013). The first wave likely came from the Indian subcontinent around 3,000 years ago, following maritime and terrestrial routes, entering Africa through today’s Egypt and the Horn of the continent. The second wave of arrival occurred during the mid-first millennium AD along Africa’s eastern coast, following the maritime trading routes. This may have brought chicken genetic diversity from as far as Southeast and East Asia (Prendergast et al. 2017). Since its introduction, domestic chicken has dispersed with human movement throughout Africa and have adapted to diverse agro-climatic conditions.

Today, backyard poultry farming constitutes an important economic activity in Ethiopia, providing both income and nutrition to poor rural households and contributing significantly to the national economy. Almost 97% of the country’s total poultry meat and egg production comes from backyard farming of chickens by small-holder farmers (Shapiro et al. 2017). Such backyard farming still relies predominantly on indigenous breeds, characterized by their tolerance to various local environmental challenges (e.g., extreme climatic conditions such as temperature and precipitation, disease, and predation) and their ability to forage for food (Getu 2014; Shapiro et al. 2017; Bettridge et al. 2018). In the absence of any management practices or breed improvement initiatives, the productivity of these indigenous chickens, however, is quite poor compared with commercial breeds raised under managed farming conditions. Elucidating the genetic basis of local adaptation of these birds will have important implications for sustainable improvement of poultry production.

Despite the observational knowledge that African indigenous chickens are adapted to their harsh environmental conditions, the genetic mechanisms underlying these adaptations are still largely unknown (Muchadeyi and Dzomba 2017). Likewise, little effort has been made to dissect livestock ecosystems to identify major environmental factors that trigger adaptive response (Muchadeyi and Dzomba 2017). Conventionally, environment−genome adaptation studies have focused on the adaptation to an inferred specific environmental stressor, for example, high altitude or heat stress (Zhang et al. 2016; Cedraz et al. 2017), or in a single ecotype (Elbeltagy et al. 2019; Walugembe et al. 2019), without analyzing the environmental stressors of the considered agro-ecology. In the present study, we are adopting a powerful integrative approach—combining ecological niche modeling (ENM) with genomic analyses (selection signature and genotype−environment association) to first dissect the environmental drivers of local adaptation and then to investigate their impact on the genome. We apply this approach across ecotypes of Ethiopian indigenous chicken populations.

## Results

### Genomic Diversity of Ethiopian Indigenous Chickens

Genomic data for the present study originated from the whole-genome sequencing (WGS) of 245 Ethiopian indigenous chicken samples from 25 different populations representing diverse agro-climatic conditions (fig. 1*A* and *B*; supplementary table S1, Supplementary Material online). Analysis of the WGS data detected 19.5 M SNPs, of which around 29% are novel. The genetic diversity of the populations is similar, with 10–12 M SNPs detected per population and a mean genome nucleotide diversity (*π*, based on individual sites) between 0.28 and 0.34. After applying stringent quality filtration, we used 14 M autosomal SNPs and 238 individuals for all downstream genomic analyses (see Materials and Methods).

Principal component analysis (PCA) based on the filtered variants reveals the structure and relatedness of the 25 populations (fig. 1*C*). Only the Hugub and Jarso populations from the Rift Valley slope in eastern Ethiopia are clearly separated from the other populations whereas only a weak substructuring based on geographic closeness is generally observed among the rest of the populations. Admixture analysis conforms to this result by showing contributions from three ancestral gene pools, with Hugub and Jarso having a major contribution from a single gene pool, which has a minor presence in the other populations (fig. 1*D*;supplementary fig. S1, Supplementary Material online). *Fst* analysis across all 25 populations shows a weighted *Fst* of only 0.04, implying a very low level of population differentiation (see supplementary fig. S2 for pairwise *Fst* between populations, Supplementary Material online).

### ENM Reveals the Environmental Diversity of Ethiopian Chicken Habitats and Identifies Important Environmental Drivers of Local Adaptation

ENM is a powerful tool for predicting the distribution of a species based on the environmental conditions of the species’ known occurrence locations. The distribution models were built using the maximum entropy algorithm implemented by MaxEnt (Phillips et al. 2006) with data on 34 different agro-climatic variables at 250 geographic data points (10 per population). These were considered as “occurrence data.” Moreover, 10,000 geographic data points from the remaining of Ethiopia were included as background points, against which the occurrence data could be projected to create population-specific environmental suitability maps. The agro-climatic variables included 21 climatic parameters, 8 soil properties, 4 vegetation parameters, and elevation data from public databases (supplementary table S2, Supplementary Material online). These variables were chosen considering their biological relevance for scavenging chickens, for example, climatic variables and elevation are expected to affect physiological tolerance of chicken, soil variables likely influence the type and abundance of food, and the vegetation parameters may affect both food availability and exposure to predation. Accordingly, these variables were considered as proxies of environmental selection pressures.

In the first step of ENM, we removed variables which are highly correlated (*r*_s_ > 0.6; except one from each correlated group) and/or with low contribution in explaining the Ethiopian chicken ecosystems (<4%) (supplementary figs. S3 and S4, Supplementary Material online). It retained only eight variables: The minimum temperature of the coldest month of a year (minTemp), precipitation seasonality which represents the variation in precipitation across a year (precSeasonality), precipitation in the wettest quarter (precWQ) of a year, precipitation in the driest quarter of a year (precDQ), soil organic carbon content (SoilOrgC), grass/shrub cover (Grassland), proportion of cultivated land (LandUse), and the dominant cultivated crop in an area (Crop_dominance). Upon further checking, the Grassland variable was removed as it showed high multicollinearity (Variance Inflation factor > 7) with LandUse. We also removed Crop_dominance because of ambiguity and possible erroneous categorization of some of the data points based on visual examination in Google Earth.

**Fig. 2.**
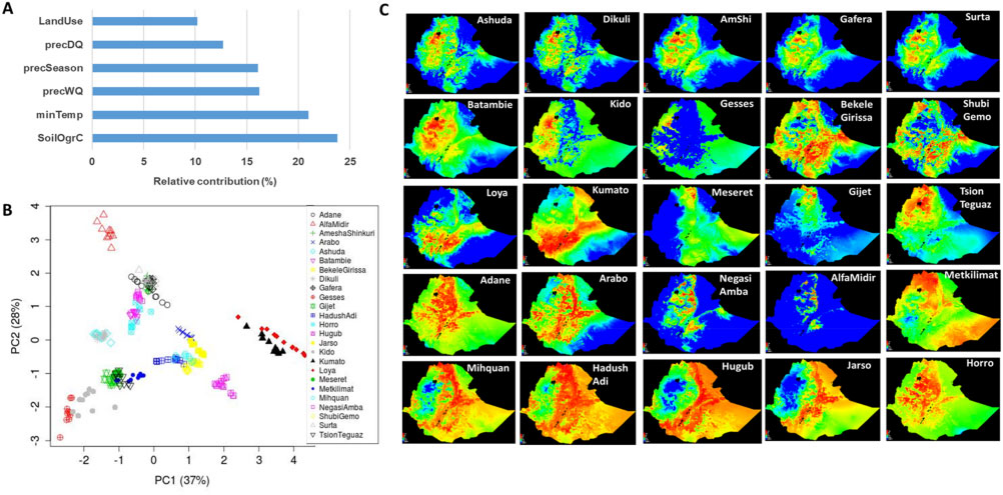
(*A*) Relative contribution of the six environmental variables selected based on Ecological Niche Modeling (ENM); (*B*) PCA plots showing the distribution of the 25 Ethiopian chicken populations in the environmental space provided by the six selected environmental parameters; (*C*) suitability maps of the 25 Ethiopian chicken populations produced by ENM using six selected environmental variables. Hotter colors (toward red spectrum) indicate more suitable conditions.

The final model with the six selected variables produced a refined estimate of the relative contribution of the variables (fig. 2*A*). SoilOrgC shows the largest individual contribution (24%), followed by the minTemp (21%), whereas LandUse has the smallest contribution (10%). The three precipitation variables (precWQ, precDQ, and precSeasonality) show a combined contribution of 45%. PCA based on these six variables spread the populations in the environmental space, showing large heterogeneity in Ethiopian agro-climatic conditions and supporting the importance of these variables as environmental drivers of adaptation (fig. 2*B*). Geographically close populations are generally positioned close to each other with some notable exceptions, for example, Alfa Midir/Negasi Amba and Arabo/Adane are geographically close to each other but distant in the environmental space. In contrast, Arabo and Jarso appear close to each other in the environmental space even though they are geographically distant (409 km; figs. 1 and 2*B*). These outlier pairs show the drastic change in Ethiopian climate and landscape even within short-geographic distances. The environmental diversity of Ethiopia is further illustrated by the environmental suitability maps (fig. 2*C*), which describe how similar is the environment across the country for each of the sampled populations.

### Genomic Analyses Identify Candidate Loci for Environmental Adaptation

With the identification of the important environmental drivers of selection, our next goal was to determine the genetic basis of adaptation to these factors. Two types of analyses were performed: 1) Selection Signature Analysis (SSA) by comparing extreme groups of populations (Low vs. High) for each environmental predictor (table 1; supplementary fig. S7, Supplementary Material online), using *Fst* (Weir and Cockerham 1984) and XPEHH (Sabeti et al. 2007) approaches with overlapping sliding windows (20 kb size with 10 kb step) and 2) Genotype−Environment Association (GEA) using redundancy analysis (RDA), a multivariate linear regression approach that can simultaneously analyze many loci to detect weak multilocus signatures of selection (Forester 2019). RDA was chosen over other GEA methods for its robust performance across different sample sizes, levels of population structure, and demographic histories.

**Table 1.** Summary Table Describing the Low and High Groups and Selection Signatures Results from Different Environmental Analyses.

Environmental Variables	Low Group Populations and Environmental Stats (Mean ± SD)	High Group Populations and Environmental Stats (Mean ± SD)	No. of Selective Sweep Regions (SRs)	Candidate Genes (Common XPEHH and Fst candidates; SSA and RDA candidates^a^)
Minimum temperature of the coldest month (minTemp)	AlfaMidir, NegasiAmba (*n* = 20) Min.T (°C): 1.83 ± 1.07 Max.T (°C): 20.61 ± 1.20 Elevation (m.a.s.l.): 3219 ± 192	Hugub, Mihquan (*n* = 20) Min.T (°C): 12.67 ± 0.92 Max.T (°C): 36.11 ± 1.18 Elevation (m.a.s.l.): 1077 ± 276	114	209 (10; 2)
Precipitation of the wettest quarter (precWQ)	Hugub, Jarso (*n* = 24) 314.05 ± 40.58 mm/m^2^	Gafera, Gesses (*n* = 19) 1088.65 ± 18.28 mm/m^2^	107	150 (0; 1)
Precipitation of the driest quarter (precDQ)	Gijet, Kido (*n* = 18) 9.40 ± 0.97 mm/m^2^	Kumato, Loya (*n* = 19) 120.90 ± 15.80 mm/m^2^	168	217 (10; 3)
Precipitation seasonality (precSeasonality)	Loya, Kumato (*n* = 19) 47.8 ± 3.62 mm/m^2^	Meseret, Gijet (*n* = 19) 141.20 ± 2.97 mm/m^2^	152	193 (4; 3)
Soil Organic Carbon (SoilOrgC)	Loya, Kumato (*n* = 19) 71.7 ± 13.52 g/kg at depth of 0 m	AlfaMidir, Adane (*n* = 20) 145.80 ± 7.49 g/kg at depth of 0 m	145	219 (7; 9)
LandUse	Gesses, Kido (*n* = 18) 1.28 ± 1.65 (%)	Meseret, AlfaMidir (*n* = 20) 39.56 ± 1.67 (%)	157	190 (7; 2)

a Common genes between one of the SSA approaches and RDA; none of the candidate genes were commonly detected by all three approaches (XPEHH, *FST* and RDA).

SSA windows with empirical *P-*value < 0.01 were considered as putative selective sweeps for a standardized *Fst* (*ZFst*) > 5 or an absolute standardized XPEHH (|XPEHH_std|) > 3 (supplementary figs. S9 and S10, Supplementary Material online). Moreover, since the positive and negative values of XPEHH indicate directionality of selection, all SNPs within a XPEHH-based candidate window needed to show the same directionality. For *Fst*-based candidate windows, we determined the direction of selection based on which group (Low or High) had the lower value of pooled heterozygosity (*Hp*) (Rubin et al. 2010), and/or based on the signs of the XPEHH value for the corresponding windows (see Materials and Methods).

**Fig. 3.**
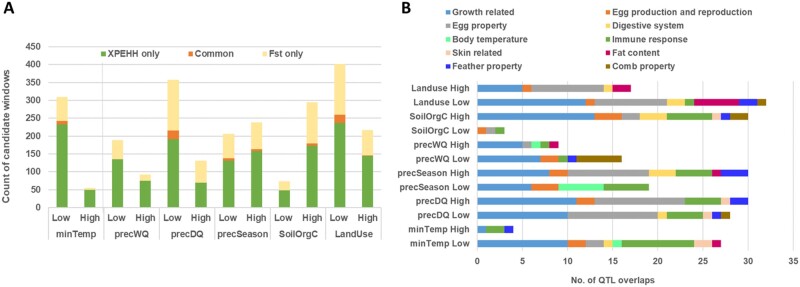
(*A*) Stacked bar plot showing the split of candidate sweep windows based on Low/High groups and detection methods; (*B*) overlap of candidate genes with known QTLs from chicken QTLdb.

Across the different environmental analyses, we observe a weak positive correlation between the *Fst* and the XPEHH results (*r_s_* = 0.22−0.34, *P *<* *2.2e−16). A similar observation has also been reported in previous studies (Ma et al. 2015). Depending on the environmental variables, *Fst* identified 71−237 and XPEHH identified 210−405 windows above the assigned thresholds (fig. 3*A*). Selective sweep windows common to both analyses were considered as our strongest candidates. The number of common windows ranged from 6 (SoilOrgC variable) to 24 (precDQ and LandUse variables) (fig. 3*A*), but none was found for the precWQ variable (fig. 3A, supplementary fig. S12, Supplementary Material online). After merging adjacent windows, 107−168 selective Sweep Regions (SRs) of 20−550 kb size range were obtained (table 1). Around 76−90% of the detected SRs overlap with genes (supplementary table S3, Supplementary Material online). The SRs harbor a large number of SNPs (∼62,000 to ∼101,000), but only 1.5−8% show a large difference in allele frequency (dAAF > 0.5), and only a handful of these (*n* = 1−35) belong to a potentially functional category (nonsynonymous, splicing, and ncRNA exonic) (supplementary table S4, Supplementary Material online). Hypergeometric tests show that intergenic SNPs are over-represented among those with large dAAF, whereas SNPs within genes (nonsynonymous, intronic, and UTR) are under-represented (*P *<* *7.34e−09). The predominance of intergenic SNPs among high-frequency variants may indicate that regulatory regions play major roles in adaptation traits. However, it is also possible that many of the intergenic variants are actually neutral but were easily hitchhiked to high frequency with causal variants without having any physiological consequence. Many of the SRs overlap with known QTLs (ChickenQTLdb), suggesting the affected phenotypes (fig. 3*B*;supplementary table S5, Supplementary Material online).

**Fig. 4.**
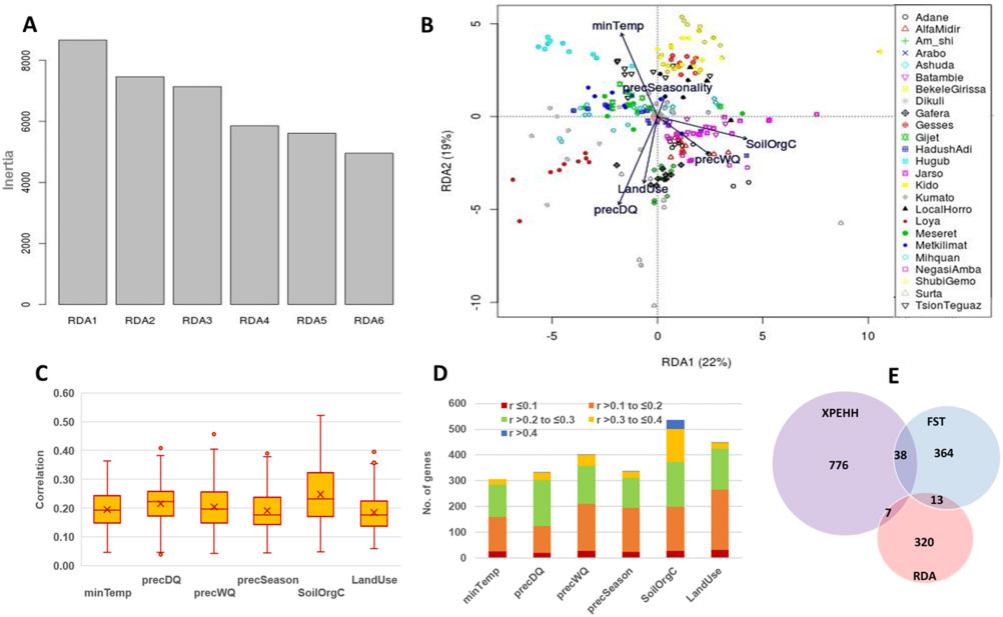
(*A*) Variance explained by RDA axes; (*B*) PCA plot based on RDA axes 1 and 2; (*C*) Box plots showing the distribution of correlation values of outlier SNPs associated with different environmental predictors; (*D*) stacked bar graph showing number of genes linked to RDA outlier SNPs and their split based on environmental correlation; only genes (with *r* ≥ 0.3) were finally considered as candidates; (*E*) Venn diagram showing overlaps of candidate genes between selection signatures and RDA analyses.

RDA was performed using a set of genome-wide LD-pruned SNPs (*n* = 1,210,311) as response variables and the six environmental predictors as explanatory variables. Conditioning on geography (latitude and longitude) was applied to correct for spatial autocorrelation and neutral population genetic structure arising from geographic proximity (Rellstab et al. 2015). In addition, population structure arising from the different origins of the chicken populations (fig. 1*D*) was corrected for by partialling out ancestry-coefficients from ADMIXTURE analysis. The overall model was highly significant (permutation analysis *P*-value < 0.01), although it explained only 0.9% of the total genetic variance. This result is not unusual given that only a small proportion of the 1.2 M SNPs is expected to be associated with environmental predictors (Forester 2019). The first five of the six RDA axes were significant, explaining about 88% of the variance captured by the RDA model (fig. 4*A* and *B*). Therefore, SNPs from the two extreme ends of the loading distribution (SD > 3.5) at each significant axis were taken as outliers (*n* = 2,863 in total). Considering the strongest correlated environmental variable for each SNP, we found 361−668 outlier SNPs per environmental predictor. The correlation values were generally low to moderate, ranging between 0.04 and 0.52 (median = 0.20) (fig. 4*C*). We applied further filtration of *r *>* *0.3 to retain only those with relatively large environmental correlation; this retained only 374 outliers. Since an LD-pruned SNP set was used in the analysis, it is quite possible that the actual causal variants were not included. We therefore identified any variants that were in complete LD with the outliers SNPs (*r^2^* = 1). This added another 96 SNPs to the candidate SNP list, taking the total to 470 (supplementary table S7, Supplementary Material online). The RDA candidate SNPs represent 320 gene−environment combinations, ranging from 21 genes for minTemp to 166 genes for SoilOrgC (fig. 4*D*). Only 4.2% (*n* = 20) of these are common with those detected in either the XPEHH or *Fst* approach but none were detected by all three methods (fig. 4*E*). The very low overlap between RDA and SSA may be attributed to two possible reasons. First, RDA was performed using LD pruned SNP set and it is likely that many environmentally associated SNPs were not actually tested. Second, RDA applies a linear regression approach. If the genotype−environment association is anything but linear, it will not be detected by RDA.

#### Adaptation to Extreme Temperatures and High Altitude

**Fig. 5.**
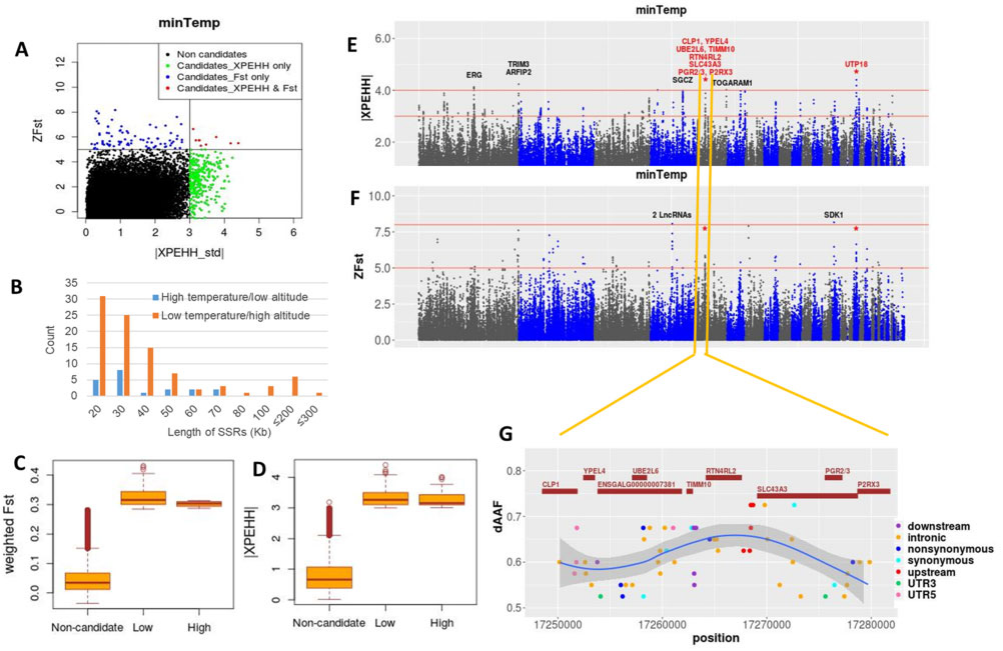
Selection signature analysis results for minTemp. (*A*) Scatter plot of standardized values of XPEHH versus *Fst*. (B) length distribution of selective Sweep Regions (SRs). (*C*, *D*) Box plots showing the distribution of *Fst* and XPEHH metrics for noncandidate and candidate windows. (*E*, *F*) Manhattan plots for the XPEHH and *Fst* analyses; common windows are marked with asterisk and gene names from common windows are shown in red. (*G*) Closer look of the common *Fst*/XPEHH region—chr5:17250000 − 17280000—with SNPs showing allele frequency difference (dAAF) > 0.5 between the Low (AlfaMidir, NegasiAmba) and High (Hugub, Mihquan) groups. Genes common between *Fst* and XPEHH are shown in red.

The minimum temperature of the coldest month of a year (minTemp) shows a strong positive correlation with the maximum temperature of the warmest month (*r*_s_ = 0.9) and a strong negative correlation (*r*_s_ < −0.91) with elevation (m.a.s.l.). Therefore, for the SSA, the Low group included two populations (Alfa Midir and Negasi Amba) living at the lowest minimum temperature, the lowest maximum temperature, and the highest altitude environments, whereas the High group included two populations (Hugub and Mihquan) living at the highest minimum temperature, highest maximum temperature and the lower-altitude environments (supplementary fig. S7, Supplementary Material online). The majority of the SRs (82%) and the strongest signals were detected in the Low group providing evidence in support of adaptation to low temperature and/or high altitude (figs. 3*A*, 5*B*, and 5*D*; supplementary table S3, Supplementary Material online). Of the 209 genes overlapping the SRs, ten genes—all in the Low group—were detected in both *Fst* and XPEHH analyses. These are considered as the most significant candidates (table 2). Nine of these genes (*CLP1*, *YPEL4*, *ENSGALG00000007381*, *UBEL6*, *TIMM10*, *RTN4RL2*, *SLC43A3*, *PGR2/3*, *P2RX3*) belong to a single SR in chr5:17250000−17280000 (fig. 5*E*−*G*), whereas the remaining one (*UTP18*) overlaps the region in chr18:5100000−5120000.

**Table 2.** Genes Detected by Both XPEHH and *Fst* in Relation to Environmental Adaptations.

Genes and Sweep Regions	Relevant Biological Functions for the Candidate Genes
***Adaptation to high altitude stresses (hypoxia, thrombosis, and cold tolerance) (Low minTemp)***	
Chr5:17230000 − 17290000^a^^,^^b^ Gene cluster: *CLP1, YPEL4*, *ENSGALG00000007381, UBEL6, TIMM10, RTN4RL2, SLC43A3, PGR2/3, P2RX3*	*CLP1*—linked to cardiovascular function and cardiac muscle hypertrophy (Espinoza-Derout et al. 2007); *YPEL4*—role in pulmonary diseases (Truong et al. 2018); *P2RX3*—blood coagulation, responses to cold, heat and hypoxia (Uniprot); *ENSGALG00000007381*—blood coagulation (Reactome); *SLC43A3*—roles in lung tissue repair and growth under oxidative stress (Furukawa et al. 2015); *PGR2/3*—immune response (Uniprot); *RTN4RL2*—roles in axon regeneration and protection of motoneurons against apoptosis (Uniprot), and upregulated in myopathy-affected condition in broiler possibly in response to hypoxia (Marchesi et al. 2019); *UBE2L6:* involved in protein ubiquitination pathway (Uniprot) ; *TIMM10*—imports transmembrane proteins into the mitochondrial inner membrane (Uniprot); downregulated in hypoxic cells (Lai et al. 2016).
Chr18:5090000 − 5190000^a^^,^^b^ *UTP18*	*UTP18*—RNA binding and involved in pre-18S rRNA processing (Uniprot); belongs to a gene-network targeted by microRNAs differentially expressed in human hypoxic cardiomyocytes cell line (Lee et al. 2018)
***Water scarcity adaptation (Low precDQ)***	
Chr3:71840000 − 73950000^a^^,^^b^ (consists of several SRs) Gene cluster: *ENSGALG00000036204* , *ENSGALG00000025686, MANEA, EPHA7*	*ENSGALG00000036204*—a lncRNA with possible cis-regulatory role on nearby genes; for example, the nearest gene is *MMS22L* with role in DNA damage repair process (Uniprot); *ENSGALG00000025686*—U6 spliceosomal RNA with possible role in post-transcriptional modification; *MANEA*—associated with panic disorder (Jensen et al. 2014); *EPHA7*—involved in many functions, for example, apoptotic process, axon guidance, brain development, ephrin receptor signaling pathway, and nephric duct morphogenesis (Uniprot)
Chr3:106430000 − 106510000^a^^,^^b^ *MSRA*	*MSRA* -Cellular protein modification, protein repair, response to oxidative stress (Uniprot).
Chr4:74170000 − 74300000^a^^,^^b^ Gene cluster: *ENSGALG00000048521*, *ENSGALG00000050078*, *ENSGALG00000046053*	These lncRNA genes possibly have cis-regulatory functions on nearby genes; a plausible nearby target is *PPARGC1A*, which is involved in many biological functions.
Chr6:25040000 − 25070000^a^^,^^b^ *SLK*	*SLK—*mediates apoptosis (Uniprot)
Chr11:18110000 − 18130000^a^^,^^b^ *BANP*	Multicellular organism development, transcriptional regulation, regulation of signal transduction (Uniprot)
***Adaptation to high precipitation seasonality: possible adaptation to water scarcity***	
Chr4:2730000 − 2750000^a^^,^^b^ *HTR2C*	Many functions, for example, behavioral fear response, regulation of appetite, regulation of corticotropin-releasing hormone secretion and nervous system processes (Uniprot).
Chr4:74280000 − 74310000^a^^,^^b^ *ENSGALG00000051573*	A lncRNA with possible cis-regulatory functions; the nearest protein coding gene is *ENSGALG00000040208*
***Possible adaptation to excess rainfall and humidity (Low precSeasonality)***	
Chr2:114000000_114100000^a^^,^^b^ *ENSGALG00000053888*	A lncRNA with possible regulatory functions; the nearest gene, *YTHDF3* has role in positive regulation of translation (Uniprot).
Chr7:35360000 − 35400000^a^^,^^b^ *CACNB4*	Calcium transport; ion transport (Uniprot)
***Scavenging adaptation to rich source of insect, worm and plant based food* (*High SoilOrgC)***	
Chr1:197230000 − 197310000^a^ Gene cluster: *HBBA*, *HBE, HBE1, HBBR*, *ENSGALG00000052767*	*HBBA, HBE, HBE1, and HBBR—*involved in heme binding, oxygen carrier activity, cellular oxidant detoxification, protein hetero-oligomerization, response to organic cyclic compound (Uniprot). *ENSGALG00000052767—*novel protein coding
Chr10:6820000 − 6870000^a^^,^^b^ Gene cluster: *THSD4*, *ENSGALG00000053176*	*THSD4*—has peptidase activity (Uniprot) with possible association with feed efficiency traits (Yao et al. 2013); *ENSGALG00000053176 is a* MiRNA with possible role in RNA silencing and post-transcriptional regulation of gene expression.
***Scavenging adaptation to low surplus of crop and grain-based food (Low LandUse)***	
Chr1:127750000 − 127980000 Gene cluster: *STS, PUDP*	*STS*—Lipid and steroid metabolism (Uniprot); *PUDP*—Nucleotide metabolic process (Uniprot).
Chr1:128960000_129350000^a^^,^^b^ *ENSGALG00000052489*	Novel protein coding gene
Chr3:34260000 − 34320000^a^^,^^b^ Gene cluster: *SMYD3, KIF26B*	*SMYD3*—Role in transcriptional regulation as a member of an RNA polymerase complex and in cellular response to dexamthasone stimulus (Uniprot); *KIF26B*—role in cell signaling (Uniprot).
Chr4:56140000 − 56170000^a^ *NDST4*	*NDST4*—Strong association with low abdominal fat content in chicken (Uniprot).
***Scavenging adaptation to crop and grain rich food (High LandUse)***	
Chr4:75690000 − 75810000^a^^,^^b^ *NCAPG*	*NCAPG—*Cell division, mitotic chromosome condensation (Uniprot).

Note.—Genes present in close proximity on the same chromosome are shown as clusters (in a few cases this involves separate sweep regions).

a Regions intersecting with highly significant FLK SNPs (*P *<* *0.01) and showing consistent pattern of allele frequency in each Low and High population.

b Regions successfully validated in a new population (i.e., overlapping significant FLK SNP showing consistent pattern of allele frequency with at least 15% difference in allele frequency between Low and High populations in the validation set).

Most of the common genes on chr5 SR can be directly related to various stress responses induced by high altitude, such as, hypoxia (Sarkar et al. 2003), thrombosis (Gambhir et al. 2014), and cold temperature (table 2). For example, *CLP1* is linked to cardiac muscle hypertrophy (Espinoza-Derout et al. 2007), *YPEL4* has a role in pulmonary diseases (Truong et al. 2018), *P2RX3* and *ENSGALG00000007381* are involved in blood coagulation (Reactome; Uniprot), and *SLC43A3* plays a possible important role in the repair and growth of the lung tissue under oxidative stress (Furukawa et al. 2015). *P2RX3* is also involved in the sensory response to cold and heat, whereas *PGR2/3* has a role in the immune response (Uniprot). Other genes, for example, *RTN4RL2,* and *TIMM10* have been found differentially expressed in cells under hypoxic conditions (Lai et al. 2016; Marchesi et al. 2019), with *RTN4RL2* protecting motor neurons against apoptosis (Uniprot), which may be an essential adaptation to high altitude-induced hypoxia. *UTP18* from chr18 SR has also been found differentially expressed in human hypoxic cardiomyocytes cell line (Lee et al. 2018) and is involved in the processing of the pre-18S ribosomal RNA (rRNA). The rRNAs are important components of ribosome—the factory for protein biosynthesis (Uniprot).

Some other notable candidates for adaptation to low temperature and/or high altitude, detected with a strong signal (*ZFst* > 8 or |XPEHH_std| > 4) but from a single approach, include *SDK1—*which regulate the dendritic spine development and synaptic connectivity (Uniprot), *TRIM3—*involved in nervous system development and critical cellular processes such as proliferation, apoptosis, and transcriptional regulation (Chen et al. 2014), and *ARFIP2—*with a role in autophagy (Uniprot).

In the High group, 25 candidate genes were found, but none were common between *Fst* and XPEHH analyses. The only gene that overlaps a strong SSA signal is *TOGARAM1* (|XPEHH_std| > 4) (fig. 5*E*), which is involved in the assembly of nonmotile cilia (Nachury and Mick 2019). These organelles are essential for cellular signal transduction and heat-shock induces their rapid loss (Prodromou et al. 2012). *TOGARAM1* may play an important adaptive role in alleviating this effect in high temperature conditions.

Ingenuity pathway analysis (IPA) of the 184 candidate genes from the Low group indicates enrichment of processes like lipid metabolism, small molecule biochemistry, and molecular transport (supplementary table S6*A*, Supplementary Material online), which are expected as hypoxia or cold-temperature stresses affect a cascade of biosynthetic and molecular processes (Chohan 1984; Hopfl et al. 2003). Low group candidates are also associated with many cardiotoxicity terms such as bradycardia, cardiac arrhythmia, heart failure, congenital heart anomaly, and cardiac enlargement—indicating involvement in hypoxia stress response. Contrarily, the genes from the High group show more enrichment for processes related to organismal growth and development (supplementary table S6*A*, Supplementary Material online). Low-group candidates overlapped with many known QTLs, including those related to skin properties, body temperature, blood parameters, abdominal fat, immune response, and production traits. Meanwhile, High group candidates showed overlap with QTLs for feather properties, disease susceptibility and immune response, and feed conversion ratio (fig. 3*B*;supplementary table S5, Supplementary Material online).

Candidate SNPs from RDA analysis are linked to only 21 genes with correlation to minTemp varying between 0.3 and 0.36. Only two of these genes have also been detected by one approach of SSA: *VMP1—*a stress-induced gene involved in the autophagy process (Uniprot), and *SEPT9—*a master transcriptional regulator of the adaptive response to hypoxia (Uniprot) (supplementary tables S7 and S8, Supplementary Material online).

#### Adaptation to Extreme Rainfall Patterns

Three of the six environmental parameters found in the ENM are related to rainfall (PrecSeasonality—variation in precipitation across the year, PrecWQ—precipitation during the wettest quarter, and PrecDQ—precipitation during the driest quarter). Precipitation variables can affect chicken biology in different ways, for example, insufficient rainfall may limit access to drinking water, whereas excessive rainfall may facilitate the spread of pathogens and parasites, challenging chicken immunity (Afrimash 2018).

Analysis of the precipitation variables provided a strong indication of adaptation to restricted water as more SRs (61% of 427 regions) and stronger signals were observed in populations where water scarcity is likely an issue; for example, in agro-ecologies with either a low rainfall (Low groups for precDQ and precWQ) or large seasonal variation in rainfall (High group for precSeasonality) (fig. 3*A*;supplementary table S3, Supplementary Material online). Prolonged water deprivation causes dehydration, which can have serious consequences on the overall physiology due to shrinkage of cells, salt-water imbalance in the body, increased osmotic pressure, renal dysfunction, and disruption of the temperature regulatory cues in the brain (Encyclopaedia of Britannica; Swayne and Radin 1991).

**Fig. 6.**
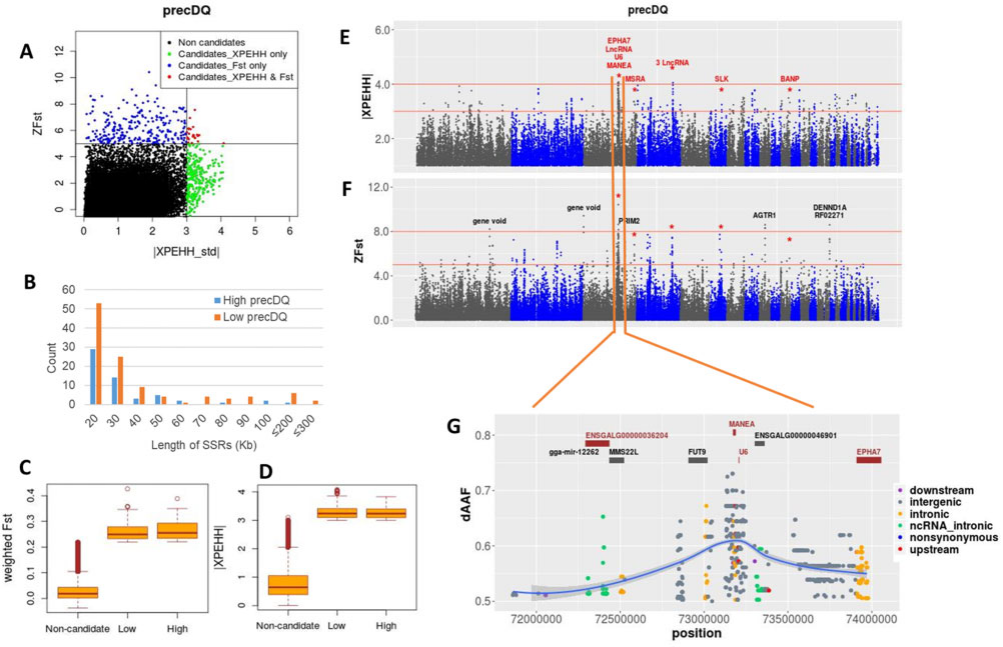
Selection signature analysis results for precDQ. (*A*) Scatter plot of standardized values of XPEHH versus *Fst*. (*B*) Length distribution of selective Sweep Regions (SRs). (*C*, *D*) Box plots showing the distribution of *Fst* and XPEHH metrics for noncandidate and candidate windows. (*E*, *F*) Manhattan plots for the XPEHH and *Fst* analyses; common windows are marked with asterisk and gene names from common windows are shown in red. (*G*) Closer look of the region—chr3:71840000 − 73950000—with SNPs showing allele frequency difference (dAAF) > 0.5 between the Low (Gijet, Kido) and High (Kumato, Loya) groups. Genes common between *Fst* and XPEHH are shown in red.

Analysis of the three precipitation variables detected 500 genes overlapping SRs, with 14 commonly identified in *Fst* and XPEHH analyses (table 2). Ten of these genes come from the precDQ Low group indicating their importance for the adaptation to dry environments where access to water may be an issue (table 2 and fig. 6). The other four genes come from the precSeasonality analysis (table 2), of which two are from the High group, indicating possible association to water restriction. Closer inspection of the High group populations (Meseret and Gijet) for PrecSeasonality confirms not only very little rainfall during the driest season (average 9.45 mm/m^2^) but also much lower rainfall in the wettest season (average 461 mm/m^2^) compared with that of all 25 populations (average 697 mm/m^2^).

The commonly detected *Fst* and XPEHH candidate genes affect many biological processes as expected from water scarcity stress, and they include a number of lncRNA genes with possible *cis*-regulatory roles on nearby genes (table 2). Figure 6*G* highlights the chr3:71840000 − 73980000 region from precDQ analysis as it harbors a cluster of several candidate genes (*n* = 8; four common between *Fst* and XPEHH) and some of the strongest signals. SNPs surrounding *MANEA* show the largest difference in alternative allele frequency (dAAF). This gene has been related to behavioral issues like panic disorder in human (Jensen et al. 2014), and it is ubiquitously expressed in many tissues, but most prominently in the urinary bladder and the thyroid (ENTREZ). Another important candidate from the same region is *EPHA7*, which is involved in many gene ontology (GO) biological processes (Uniprot) including apoptotic process, axon guidance (in response to environmental cues), brain development, ephrin receptor signaling (important in kidney physiology), and nephric duct morphogenesis.

*HTR2C* is another common candidate gene from *Fst* and XPEHH (table 2), detected from precSeasonality analysis (supplementary fig. S11, Supplementary Material online). This gene plays a crucial mediatory role in the stress-induced activation of the hypothalamic−pituitary−adrenal axis (Brummett et al. 2014). It is involved in many biological processes, including behavioral fear response, regulation of appetite, and regulation of nervous-system processes (Uniprot).

Genes that overlap with strong signals from a single approach (*ZFst* > 8 or |XPEHH_std| > 4) in relation to water scarcity include: *AGTR1*, *TMEM206*, and *ATF3. AGTR1* plays a role in the regulation of blood pressure, sodium retention by the kidney, and in kidney development (Uniprot). *TMEM206* is involved in pH-gated chloride channel activity that helps to maintain the body’s acid−base balance (Uniprot). *ATF3* is a previously reported common stress-responsive transcription factor (Uniprot; Zhao et al. 2016).

We detected two common candidate genes from the precSeasonality Low group, that is, the population showing low variation in rainfall pattern. *CACNB4* has a functional role in the calcium ion transport (Uniprot), whereas *ENSGALG00000053888* encodes a lncRNA with possible regulatory function. It is not clear what type of stress response these genes are involved with, but these may be associated with the adaptation to an environment with high ambient humidity. We observe that the relative humidity in the precSeasonality Low group (59−109%) is generally higher both in the wettest and driest quarters of a year compared with that in the High group (57−71%) (supplementary fig. S8, Supplementary Material online). Two other genes overlap with strong signals in populations experiencing greater rainfall or lower variation in annual rainfall: *PRIM2* (from the precDQ High group; *ZFst* > 8) with a role in DNA replication and *GTDC1* (from the precSeasonality Low group; |XPEHH_std| > 4) showing ubiquitous expression among tissues (Uniprot). IPA analyses of the candidate genes from both the Low and High groups (supplementary table S6*B*−*D*, Supplementary Material online) as well as the overlap of the genes with known QTLs (fig. 3*B*;supplementary table S5, Supplementary Material online) indicate their involvement in a wide range of biological processes.

The RDA analysis identified 105 candidate genes in relation to the precipitation variables, of which only seven genes were also detected by one method of SSA and a few candidates (*n* = 7) are linked to SNPs with relatively larger environmental correlation (*r *≥* *0.4) (supplementary table S8, Supplementary Material online). Many of these genes are directly involved in various stress response pathways or processes. For example, *GPC5* is involved in the regulation of the *Wnt* signaling pathway (Uniprot), which mediates stress granule assembly in cells (Lai et al. 2016); *HMGCLL1* is involved in the biosynthesis of ketone bodies that play an important role in maintaining the body’s redox homestasis in response to environmental or metabolic stressors (Rojas-Morales et al. 2020); *SLK, SLIT3*, and *PHLPP1* have involvement in apoptotic processes (Uniprot); and *GDPD1* has been reported to be upregulated under drought stress in some plant species (Kotrade et al. 2019). *GPC5* has also been found associated with renal disease (Okamoto et al. 2015). Some other genes possibly have roles in broader physiological adjustments under stressful condition, for example, *PPFIA2—*has involvement in nervous system processes, *MRPL46*—encodes a structural component of the mitochondrial ribosome, and several lncRNA genes with their potential regulation of neaby genes (supplementary table S8, Supplementary Material online).

#### Soil Organic Carbon—An Indicator of Source and Abundance of Food for Scavenging Chickens

**Fig. 7.**
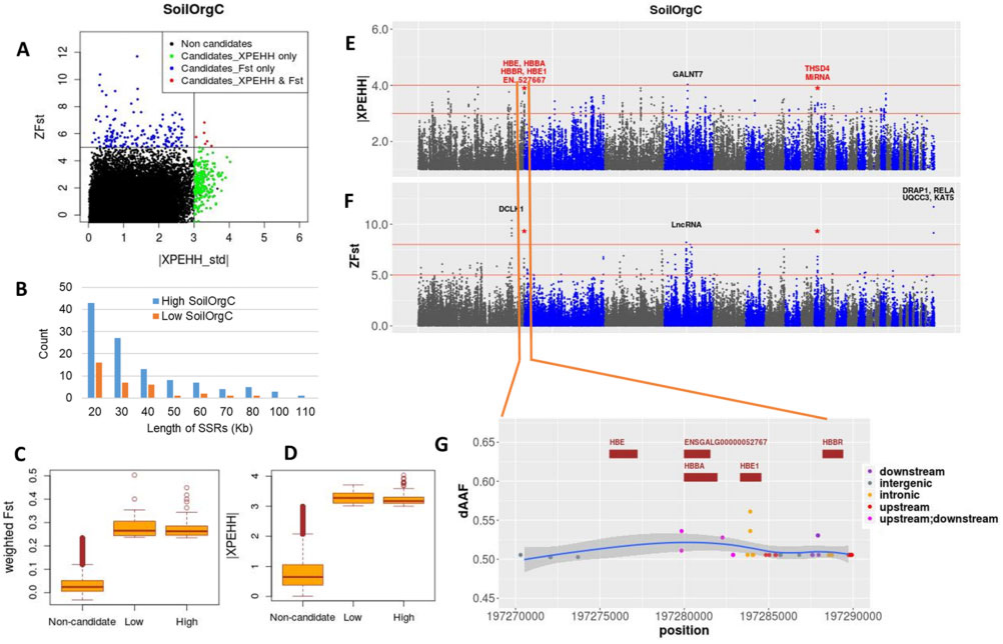
Selection signature analysis results for SoilOrgC. (*A*) Scatter plot of standardized values of XPEHH versus *Fst*. (*B*) Length distribution of selective Sweep Regions (SRs). (*C*, *D*) Box plots showing the distribution of *Fst* and XPEHH metrics for noncandidate and candidate windows. (*E*, *F*) Manhattan plots for the XPEHH and *Fst* analyses; common windows are marked with asterisk and gene names from common windows are shown in red. (*G*) Closer look of the common *Fst*/XPEHH region chr1:197270000 − 197290000 with SNPs showing allele frequency difference (dAAF) > 0.5 between the Low (Loya, Kumato) and High (Meseret, Gijet) groups. Genes common between *Fst* and XPEHH are shown in red.

Soil organic carbon (SoilOrgC) affects the nature and abundance of animal biomass in the soil. A High SoilOrgC will be characterized by the presence of many earth-dwelling organisms such as insects and worms that are excellent sources of protein-rich food for chickens. Soils rich in organic carbon also provide fertile ground for wild vegetation and for growing crops—equally important for scavenging birds. Only two chromosomal regions (chr1:197270000 − 197290000 and chr10: 6820000 − 6850000) and seven genes—all in the High SoilOrgC group—were identified in both *Fst* and XPEHH analyses (fig. 7). The SR in chr1 overlaps with a cluster of several heme-binding genes (*HBBA*, *HBE*, *HBE1*, and *HBBR*) involved in oxygen carrier activity. The *HBE1* gene is also involved in GO biological process: Response to organic cyclic compound and protein hetero-oligomerization (Uniprot). The dAAF pattern is mostly homogeneous across these genes, but a few intronic SNPs from *HBE1* show the largest dAAF (fig. 7*G*). These genes are overexpressed in broilers with severe myopathic breast muscles, possibly as a response to insufficient oxygen and oxidative stress (Pampouille et al. 2019). Accordingly, their expression may be adaptive in scavenging chickens in response to oxidative stress from food conversion in high SoilOrgC agro-ecology.

The chr10 region overlaps with two genes, *THSD4* and *ENSGALG00000053176 (miRNA)*. *THSD4* has peptidase activity, and the gene has been found in an *Fst*-based selective sweep between Red Junglefowl (RJF) and commercial birds in a previous study (Qanbari et al. 2019). *THSD4* is also a candidate gene for a feed efficiency trait in dairy cattle (Yao et al. 2013). It is likely that the peptidase activity of *THSD4* plays an essential role in the metabolism of protein-rich foods available in high SoilOrgC agro-ecologies. Located within *THSD4*, the miRNA gene may be postulated to have a regulatory effect on its expression.

Other candidates overlapping strong signals from a single approach in the High SoilOrgC group include *DCLK1* (*ZFst* > 10) with protein kinase activity and possible involvement in the nervous system and forebrain development, and *GALNT7* (|XPEHH_std| > 4), involved in carbohydrate metabolic process (Uniprot). From the Low SoilOrgC group, four genes overlapped with a strong SR on chr33 (chr33:6470000 − 6500000) (*ZFst*: 9 − 12). These include *DRAP1—*involved in transcriptional regulation (Uniprot), *RELA—*a ubiquitously present transcription factor affecting many biological processes including cell growth, immunity, and apoptosis (Uniprot), *UQCC3* which plays an important role in ATP production by mitochondria (Uniprot) and *KAT5—*regulating many biological processes including autophagy under starvation condition (Uniprot). IPA analysis shows many similar molecular and cellular functions being affected by genes from both Low and High groups but contrasting processes include Cell Death and Survival in the Low group whereas Cellular Development, and Cellular Growth and Proliferation in the High group (supplementary table S6E, Supplementary Material online), indicating the effects of abundance of food on chicken physiology.

RDA analysis has identified 166 candidate genes for SoilOrgC. Of these, 9 genes were also detected by one of the SSA methods and 36 genes showed *r *≥* *0.4 (supplementary table S8, Supplementary Material online). Many of these genes are involved in the development and processes of brain, eye, ear, and nervous system (e.g., *KIF5C, ZEB2, RAB5A, TENM3, GABRB3, MDGA1, ACVR2B, FARP1, SPARC*), and in mediating the senses of vision, smell, and taste (*ADGRA3*) — all of which are essential for successful foraging behavior (supplementary table S8, Supplementary Material online). Other important biological processes affected by the major candidates include: growth, development, and reproductive processes (*SPARC, NUMA1, CDH6, ITGA11, KIF23, PAPPA, ACVR2B, CDYL)*, metabolic processes and feed conversion (*PEX1, PAPPA, FAM13A, EPB41*) and transcriptional regulation (*HMGA2, CMSS1, CCNC, COQ3, CDYL, ETV3, PABPC1*, and several lncRNA genes).

#### Land Use Pattern is an Important Determinant of Food Abundance for Scavenging Chickens

By-products from the harvesting and processing of cultivated grains or crops are important sources of plant-based food for scavenging chickens (Tadelle et al. 2003). Interestingly, from 191 genes overlapping candidate SRs, the majority (72%) are selected in the Low LandUse group (i.e., populations living in regions with a low proportion of cultivated land), where supplementation with crops residues and grain is expected to be less. Only seven genes from five sweep regions are common to *Fst* and XPEHH analyses, of which six are from the Low group (table 2). The Low group genes include two clusters: *STS* and *PUDP* from chr1:127800000 − 127830000, and *SMYD3* and *KIF26B* from chr3:34260000 − 34280000. *STS*, with its role in lipid and steroid metabolism, is a candidate gene for growth and feed efficiency traits in cattle (Mukiibi et al. 2019), whereas *PUDP* is involved in nucleotide metabolism (Uniprot). In the second cluster, *SMYD3* is involved in transcriptional regulation and cellular response to dexamethasone (a corticosteroid hormone) that can affect appetite (Sarcev et al. 2008), and *KIF26B* has a role in cell-signaling (Uniprot). Another common gene from the Low group is *NDST4*, which has a strong association with low abdominal fat content in chicken (Zhang et al. 2012).

*NCAPG* is the only common gene between the *Fst* and XPEHH analyses in the High group (i.e., populations living in regions with a high proportion of cultivated land). This gene has been reported to be associated with various growth-related traits in beef cattle and carcass traits in chicken (Lindholm-Perry et al. 2011; Ma et al. 2019).

From the Low LandUse group, several genes overlapped with strong signaling SRs from a single approach (*ZFst* > 8 or |XPEHH_std|> 4) (supplementary fig. S13, Supplementary Material online), including *AGMO* which has roles in lipid metabolism and feed efficiency in chicken (Izadnia et al. 2019), *MED8—*involved in transcriptional regulation, and *SZT2—*involved in cellular response to amino-acid and glucose starvation (Uniprot). From the High LandUse group, notable genes overlapping with strong SR signals include *ADIPOR2—*regulating glucose and lipid metabolism, *SNX10—*with roles in gastric acid secretion, bone resorption and calcium ion homeostasis, and several genes involved in transcriptional regulation (*PRDM5, CBX3*, and *HNRNPA2B1*) (Uniprot). Some of the largest SRs were detected in LandUse analysis (300 − 550 kb), indicating relatively recent selection events. Interestingly the largest SR (chr4:25890000 − 26440000; 550 kb) was detected in the High LandUse group, possibly indicating a recent cultivation of the areas from where the chicken samples originated. This SR overlap with only lncRNA genes which likely have *cis*-regulatory functions on nearby protein coding genes, for example, the *CBR4* gene which is involved in the fatty acid biosynthesis pathway (Uniprot). Similarly, most other large SRs harbor many ncRNA genes (supplementary table S3, Supplementary Material online).

Strong candidate genes from RDA analysis (*r *≥* *0.4 or common RDA-SSA genes) are involved predominantly in nervous system processes and visual learning (*ITGB1, GABRG3, STAU2*), whereas some genes also have roles in metabolic, maintenance, and reproductive processes (*GDPD4, ITGB1*) (supplementary table S8, Supplementary Material online).

#### Shared Candidates Between Environmental Predictors

We detected between 150 and 219 genes from sweep regions in relation to each environmental predictor (table 1). Interestingly, about 15% of these genes (152 of 1008) are common to two or more environmental analyses (supplementary fig. S14, Supplementary Material online). The proportion of shared genes between environmental analyses varied between 0.3% and 14%. Although these shared genes may represent pleiotropic effects or shared pathways of stress response as observed with IPA analysis (supplementary fig. S14*C*, Supplementary Material online), the low rate of overlap in general indicates that our environmental analyses have captured different components of environmental selective pressures that in turn have shaped genomes distinctively.

In a few cases, the same gene was a candidate in both Low and High groups for the same environmental analysis, for example, *ZNF451* (precDQ), and four genes (*ADGRL3, CIS, CIR, HMGCLL1*) for the SoilOrgC parameter. These genes traverse multiple windows, with separate windows under selection in the two groups. Selection of different regulatory elements or use of alternatively spliced transcripts of the genes in the opposing groups may be responsible for the results. However, although intronic variants from these genes show generally the largest dAAFs, none are annotated as splice variants.

#### Further Validation of Sweep Regions

To further validate the detected sweep regions, addressing in particular the possible effects of demography and population structure on selection signature signal, we performed an FLK test (Bonhomme et al. 2010) on genome-wide SNPs based on the same four populations used for each environmental analysis. FLK corrects for the effects of population structure and demographic events due to drift by taking into account kinship matrix between populations before identifying loci that show outstanding variations in frequency. The outlier SNPs detected in this approach were then intersected with the sweeps from XPEHH and *Fst* analyses. The majority of the 843 SRs (*n* = 807; 96%) overlap with at least one FLK-outlier (*P* < 0.05) (see supplementary table S3, Supplementary Material online). Taking the most significant FLK outlier overlapping each of these regions, we checked for their consistency in allele frequency in the Low and High group populations (e.g., if the allele frequency of the SNPs is higher in each population from one extreme group compared with the populations in the other group). We find this holds true in 79% of the cases (*n* = 635 of 807 SRs). All the strong candidate sweep regions discussed above, except two (an XPEHH region containing *TOGARAM1* gene in minTemp analysis and a common XPEHH-*FST* region containing *STS* and PUDP genes in LandUse analysis), were validated by this approach.

Last but not least, we wanted to confirm our sweep regions in independent populations. For this, we created a validation set—for each environmental variable—by taking a new environmentally similar population from one group (e.g., Low group) while taking the most extreme population (that was originally used) from the other group. We then checked for the consistency of allele frequency direction (as observed in the original sets) of the most significant FLK outlier representing a sweep region in the validation set. About 87% of SRs validated above (552 of 635) were found consistent in an independent population (see supplementary table S3, Supplementary Material online). Again all the strong candidates discussed in the above sections that passed the first validation, could also be validated in an independent population, except two regions (see table 2 and supplementary table S3 for the validation status of the SRs, Supplementary Material online).

## Discussion

Changes in climatic condition, production environment, and customer demand are shifting the breeding goals for poultry from merely improving production traits to also incorporating welfare, environmental resilience, and disease resistance traits (Muchadeyi and Dzomba 2017). Free range, organic production is also gaining customer preference. Indigenous breeds, with their many adaptive traits including foraging ability, represent excellent genetic resources that can be harnessed for breeding improvement to cater for these emerging needs. Dissecting the genetic basis of environmental adaptation, however, is difficult due to the complexity of agro-climatic stressors posing as selection pressures. To address this issue, our study applied a powerful interdisciplinary approach to first, disentangle and identify the important agro-climatic drivers of adaptation from a large array of environmental parameters and then to identify the associated candidate regions, genes, and variants using multiple complementary genomic approaches. In the *Fst* and XPEHH-based selection signature analyses, we compared extreme populations in relation with single environmental parameters. Contrarily, in the RDA analysis we looked for genotype−environment association across the continuum of environmental parameters by simultaneously fitting all variables in the model. This is the first study, to the best of our knowledge, to perform such rigorous and comprehensive analysis on a livestock species and more specifically on chicken.

Our study has detected strong adaptive signals in relation to high altitude-induced stresses (viz. hypoxia, thrombosis, and cold temperature), water scarcity stress due to dry season, and for the first time, has identified the environmental proxies affecting scavenging conditions and associated genetic adaptations, reflecting the impact of the nature and abundance of food on foraging chickens. Interestingly, however, we did not find notable strong signals for heat stress adaptation. It is possibly a legacy of the origin and history of African indigenous chickens, whose ancestors were native inhabitants of tropical South and South-East Asia and were already adapted to the hot and humid climates of these regions (Pitt et al. 2016). However, when introduced to high elevation regions of Africa, chickens had to rapidly adapt to the challenges of their new environment, explaining why we detected more prominent selection signals in relation to altitude-induced stresses instead of heat-tolerance. As a proof of concept, we specifically looked at two heat shock genes, *HSP70* and *HSP90*, which were found in a recent study to be overexpressed in Brazilian backyard tropical chickens compared with commercial birds (Cedraz et al. 2017). We find very little allele frequency difference for SNPs across these two genes between the High and Low temperature groups. Although a number of SNPs (*n* = 19, all from *HSP90*) have reached near fixation (AAF > 0.9) in the High temperature group, these variants are also present at similar high frequency in other Ethiopian chicken populations including the Low temperature group. This suggests that the same haplotype of the above genes is present across all Ethiopian populations. Given little genetic variation in these genes, it is highly likely that their expression patterns are regulated by epigenetic elements. This gains support from the observation in previous studies that thermal manipulation during chicken embryonic development improves thermo-tolerance later in life (Nassar and Elsherif 2018). Further studies combining transcriptomic and epigenetic data, however, will be required to establish this.

Comparison of our results with the few other available adaptation studies on chicken found little overlap among the candidate genes. For instance, similar to our study, Zhang et al. (2016) reported candidate genes affecting cardiovascular and respiratory systems, and immune responses in Tibetan highland chickens as adaptations to hypoxia, but none are common with our set. This result may be attributed to a number of factors. First, rapid adaptation often works on the “standing variations” in a population (Rees et al. 2020). Different demographic history of the African and Tibetan chickens may have offered distinct standing variations for the natural selection to work on. As a result, even though the adaptive responses were similar in both instances, the associated genes were different. Moreover, epistatic interactions and pleiotropic effects of genes may favor selection of one gene over another in different geographic areas (Ostman et al. 2012). Selection of different genes in different populations in relation to high altitude has also been observed in human studies (Rees et al. 2020). These results illustrate the plasticity of the genome in its response to environmental selection pressures.

Among the identified candidate genes for each environmental predictor, only a few may be considered as strong candidates, as those were detected by multiple approaches or coincided with extreme signals from a single approach. It suggests that these environmental adaptations are predominantly under oligogenic control—an observation supported by other studies as well (Bell 2009; Rees et al. 2020). Also, in many instances, we have found important biologically relevant candidate genes residing in the same selection region or at close proximity; for example, we detected nine major candidate genes associated with hypoxia, low temperature, and thrombosis from a single SR in chr5, cluster of eight genes from chr3 in relation to water scarcity stress, and cluster of four heme-binding genes detected in chr1 from the SoilOrgC analysis. Such gene clusters may actually be at the root of rapid adaptation to extreme environment by being under the genetic control of one or a few regulatory variants only. Although further deeper investigation is required, including the identification of the causative mutations, such results provide a new framework to explain the rapid adaptation and success of an ubiquitously adapted species like chicken to different agro-ecologies. This finding has direct implication for achieving fast and sustainable improvements in new breeding programs aiming to produce chicken lines that will be both productive and well-adapted to the African backyard farming system. With predominantly oligogenic regulation of adaptive traits, the best option for achieving genetic progress would be to combine Genomic Estimated Breeding Value (GEBV) for production traits with a targeted marker-associated selection for environmentally adaptive regions of the genome.

Our study is reporting a low level of overlaps between SSA and RDA results, with none of the strongly supported candidate sweep regions (e.g., regions common to XPEHH and *Fst*) intersecting with RDA outliers. Also, most RDA outliers showed only low to moderate levels of environmental correlation (generally *r *<* *0.5). In a context of extreme environments, a strong selection for adaptation will be expected, leading to rapid fixation or near fixation of haplotypes. Such a signal of positive selection will be detected by XPEHH and *Fst* analyses. On the opposite, RDA can only detect candidates that are showing linear association across an environmental gradient and not any nonlinear response that may have occurred under extreme conditions. Much evidence of nonlinear gene−environment interaction (G × E) has been observed in agricultural and livestock species as well as in human disease studies (Ma et al. 2011; Yang 2014; Carvalheiro et al. 2019). Yang (2014) indeed suggests that linear functions would account for a small portion of G × E variation when a wide range of environmental conditions are tested, which is the case for the Ethiopian landscape, with its highly diverse agro-ecological niches for chickens. Therefore, the SSA and RDA approaches used in the present study should be seen as complementary.

Although ENM has been employed extensively for different purposes in wild species and plants, its application in livestock has been negligible. Ours is among the very few early studies that have employed ENM for dissecting livestock ecosystems. This study specifically exemplifies the use of ENM as a powerful predictive tool for adaptation analysis in livestock. Uniquely, it allowed us to first identify the key environmental drivers of selection pressure and then to investigate the corresponding genome responses, instead of the contrary approach commonly applied in animal adaptation studies. Apart from climatic and geographic factors, for example, temperature, elevation, and rainfall, our study identifies other environmental factors like soil and land use properties as important parameters in the ecosystems of foraging chickens. For the first time we show that these variables can be considered as proxies of scavenging conditions of indigenous chickens. We envisage many other different applications of ENM for livestock. For example, environmental characterization of populations, as shown in the present study, can be the basis for characterizing or even defining livestock ecotypes. Along with the agro-climatic data, any other environmental variable (e.g., epidemiological) may be included (Vajana et al. 2018). Habitat suitability mapping can be a useful approach for predicting suitable areas for introducing exotic breeds (Lozano-Jaramillo et al. 2019) or for predicting the impact of climatic change on livestock habitats. These can be extremely valuable in conservation of important livestock genetic resources to meet future demand.

## Materials and Methods

### Sampling Design

Chicken samples analyzed here consisted of 224 birds from 23 populations (villages or Kebeles) collected for this study and 21 samples from two other populations (Horro and Jarso) from a previous study (Lawal et al. 2018). The sampled populations represent different agro-ecological zones (HarvestChoice 2015) distributed across 13 districts of Ethiopia and six of the nine national regional states (supplementary table S1, Supplementary Material online). Blood samples from 8 to 10 chickens per population were collected with the logistical support and agreement of the Ethiopian Ministry of Agriculture and Ethiopian Institute of Agricultural Research (EIAR). All animal works were approved by the Institutional Animal Care and Use Committee of the International Livestock Research Institute (IREC2017-26). Geographic coordinates (latitude and longitude) of the sampling villages were recorded, providing the entry points for the collection of environmental data for ENM.

### Environmental Data

Environmental data across the Ethiopian landscape were obtained from several public databases at a spatial resolution of 30 arc-seconds (∼1 km^2^) (see supplementary table S2 and supplementary methods for details, Supplementary Material online).

For each population, we originally recorded a single central coordinate (e.g., market place) in the village. However, to capture the environmental condition for the village, we selected nine additional geographic coordinates in separate grids surrounding the actual recorded location for each population. These grids were within 1.2 km from each other and were located using Google Earth Pro v7.3. The environmental data were extracted using the “raster” R package, resulting in a total of 250 coordinate points from all 25 populations and these were considered as “occurrence points” for ENM.

### Ecological Niche Modeling

ENM was performed using MaxEnt v3.4.1 (Phillips et al. 2006). The R package “MaxentVariableSelection” (Jueterbock et al. 2016) was used to shortlist the environmental variables. The optimized model parameters used for ENM included three Feature Classes, viz. Hinge, Quadratic and Product and a Regularization Multiplier value of 3.5 (supplementary fig. S5 and further details in supplementary method, Supplementary Material online). The predictive power of the models was assessed using the Area Under ROC Curve (AUC) values (supplementary fig. S6*A*, Supplementary Material online) and the importance of the variables in the test and training data was assessed with a jackknife assay (supplementary fig. S6*B*, Supplementary Material online). Habitat suitability maps were generated using MaxEnt’s cumulative output.

### WGS and Data Processing

WGS was performed on an Illumina HiSeqX platform in paired-end mode with a read length of 150 bp and average coverage of ∼40×. Sequence reads were mapped against the GRCg6a reference assembly using BWA-mem v.0.7.15-r1140 (Li 2013). Variant calling was performed following the GATK v3.4 best practice protocol (Broad Institute 2015) involving the Haplotype Caller method and Joint Genotyping of all samples together. Initial variant filtration was performed using the VQSR approach (Haas 2015) in GTAK using 1 M validated SNPs (Kranis et al. 2013) and ∼20 M known chicken SNPs (Ensembl release 92).

Genomic analyses were performed using only autosomal variants. Individuals with high relatedness (>0.9) were removed based on relatedness calculation in VCFtools v0.1.15 (Danecek et al. 2011). SNPs that did not pass the following criteria were excluded: Genotype quality ≥15, depth of coverage ≥3, and missing genotype rate <20%. Nucleotide diversity was calculated using the –site-pi option in VCFtools. PCA was performed using the Eigenstrat method in Eigensoft v6.1.4 software (Price et al. 2006). Admixture analysis was performed in ADMIXTURE programme v1.3.0 (Alexander et al. 2009) with *K* values 1−5. The best *K* value was chosen based on the cross-validation method (supplementary fig. S1, Supplementary Material online).

### Selection Signature Analysis

Extreme Low and High groups of populations were chosen by ranking the 25 populations for each environmental parameter and selecting 2 populations from each end of the gradations. Combining two populations per group was a deliberate attempt to mitigate any potential bias from population structure and demographic events (see supplementary method “Mitigating the effects of population structure and demographic events on selection signature,” Supplementary Material online).

SSA was performed in overlapping sliding windows with at least 10 SNPs. *Fst* analyses were performed in VCFtools using the Weir and Cockerham approach (Weir and Cockerham 1984). The weighted *Fst* values were standardized (*ZFst*) to allow setting the same threshold across analyses. XPEHH analyses were carried out using the Hapbin package (Maclean et al. 2015) after removing SNPs with missing genotypes. XPEHH analyses were first performed for individual SNPs and then mean values were calculated within windows for both the standardized XPEHH (XPEHH_std) and the absolute value of XPEHH_std. Fewer windows were analyzed in XPEHH (91.2K−91.6K) compared with *Fst* (92.4K−92.5K) due to the application of some extra filtration steps (see supplementary methods for details, Supplementary Material online). Empirical *P*-values were calculated for both *Fst* and XPEHH by ranking the windows based on each metric.

Pooled heterozygosity (*Hp*) (Rubin et al. 2010) in windows was calculated for Low and High groups separately to provide an extra source of support for the directionality of selection. *Hp* results were consulted when directionality could not be established from the XPEHH result unambiguously or because those windows were not analyzed in XPEHH. Windows were removed from the downstream analysis when the directionality could not be resolved.

As a further validation approach, an FLK test was performed on genome-wide SNPs using the package hapFLK v1.4 without specifying any outgroup.

### Redundancy Analysis

RDA was performed in Vegan v2.5-4 in R (Oksanen 2015) following (Forester 2019) (further details are in supplementary method and codes used are in supplementary file “SI_code_and_results_RDA,” Supplementary Material online).

### Functional Interpretation of SRs, Candidate Genes, and Variants

The putative SRs were intersected with known genes from Ensembl (release 98) using Bedtools v2.26 (Quinlan and Kindlon 2017). Candidate genes were checked for their overlap with known chicken QTLs (ChickenQTLdb). Only significant QTLs with size <1 Mb were considered. Candidate genes were also analyzed for their molecular and cellular functions, and physiological processes using IPA (QIAGEN Inc.). SNPs within SRs were annotated using ANNOVAR (Wang et al. 2010). Hypergeometric tests for under- or over-representation of the SNP annotation categories were performed with R “phyper” function.

## Supplementary Material

Supplementary data are available at *Molecular Biology and Evolution* online.

## Supplementary Material

msab156_Supplementary_Data
