# Supplementary material for: Integrated Environmental and Genomic Analysis Reveals the Drivers of Local Adaptation in African Indigenous Chickens
Source: Mol Biol Evol. 2021 May 22;38(10):4268–85. doi: 10.1093/molbev/msab156 (PMC8476150; doi:10.1093/molbev/msab156)

This file shows the command used for running RDA and extracting results.
Lines in red font shows commands, "#" shows comments, and the rest are results.
The analysis is based on the tutorial by Forester (2018):
https://popgen.nescent.org/2018-03-27_RDA_GEA.html

Load package and data and check data

# Add vagan library

```
library(vegan)
```

# Read genotype and environmental data files and save as dataframes

```
geno <- read.table("eth_ldpruned_WGsnps_genoAltDosage", header=TRUE, sep=" ")
env <- read.table ("eth_envData_6vars_latLong_ancestryCoeff", header = TRUE, sep = "\t")
```

# Check genotype data

```
dim(geno)
[1]    238    1210312

str(geno)
'data.frame':      238 obs. of     1210312 variables:
 $ sample              : Factor w/ 238 levels "ABB-2H_Pink",..: 1 2 3 4 5 6 7 8 9 10 ...
 $ chr1_17206       : int    2 1 0 0 1 0 0 0 0 0 ...
 $ chr1_17316       : int    0 0 0 0 0 0 0 0 0 1 ...
 $ chr1_19648       : int    0 1 0 0 0 1 1 1 0 1 ...
 $ chr1_19731       : int    1 1 0 0 0 0 0 0 0 0 ...
```

# Convert the sample name from factor to character.

```
geno$sample <- as.character(geno$sample)
```

# Check environmental data

```
dim(env)
[1] 238    13

str(env)
'data.frame':      238 obs. of     13 variables:
 $ sample                : Factor w/ 238 levels "ABB-2H_Pink",..: 1 2 3 4 5 6 7 8 9 10 ...
 $ long                  : num    36.9 36.9 36.9 36.9 36.9 ...
 $ lat                  : num    10.7 10.7 10.7 10.7 10.7 ...
 $ k1                    : num    0.02648 0.00736 0.0671 0.08253 0.02406 ...
 $ k2                    : num    0.279 0.343 0.256 0.334 0.501 ...
 $ k3                    : num    0.694 0.649 0.676 0.583 0.475 ...
 $ minTemp              : int    73 74 74 71 72 74 72 74 52 52 ...
 $ precSeasonality: int    92 94 94 92 92 93 92 93 86 86 ...
 $ precWQ               : int    1002 1002 1000 1003 1003 1002 1001 999 1062 1062 ...
 $ precDQ               : int    48 45 45 50 48 46 48 45 92 92 ...
 $ SoilOrgC            : int    134 125 134 130 130 127 133 135 133 133 ...
 $ LandUse             : num    29.7 25.3 26.8 29 29.2 ...
 $ Kebele              : Factor w/ 25 levels "Adane","AlfaMidir",..: 6 6 6 6 6 6 6 6 24 24 ...
```

# Convert the sample name from factor to character.

env$sample <- as.character(env$sample)

# Check if the order of genotype and environmental data are same
identical(rownames(geno), rownames(env))
[1] TRUE

Running RDA

# First extract only the data columns from geno dataframe.
If this is not done then error message appears saying "Error in colMeans(x, na.rm = TRUE) : 'x' must be numeric"

```
geno_onlyNumeric <- geno[,2:1210312]
str(geno_onlyNumeric)
```

```
'data.frame':	238 obs. of	1210311 variables:
 $ chr1_17206	: int	2 1 0 0 1 0 0 0 0 0 ...
 $ chr1_17316	: int	0 0 0 0 0 0 0 0 0 1 ...
 $ chr1_19648	: int	0 1 0 0 0 1 1 1 0 1 ...
```

# Run partial RDA by conditioning data on lat and long

```
eth.rda_partial <- rda(geno_onlyNumeric ~ minTemp + precSeasonality + precWQ + precDQ + SoilOrgC +
LandUse + Condition(lat+long+k1+ k2+k3), data=env, scale=T)
```

# Summary result of RDA

```
eth.rda_partial
```

```
Call: rda(formula = geno_onlyNumeric ~ minTemp + precSeasonality +
precWQ + precDQ + SoilOrgC + LandUse + Condition(lat + long + k1 + k2 +
k3), data = env, scale = T)

                    Inertia Proportion Rank
Total            1.210e+06   1.000e+00
Conditional      8.011e+04   6.619e-02     5
Constrained      3.969e+04   3.279e-02     6
Unconstrained 1.091e+06   9.010e-01   226
Inertia is correlations

Eigenvalues for constrained axes:
RDA1 RDA2 RDA3 RDA4 RDA5 RDA6
8664 7463 7140 5854 5610 4956

Eigenvalues for unconstrained axes:
   PC1     PC2     PC3     PC4     PC5     PC6     PC7     PC8
12735 12276    9150    8703    8600    8107    8032    7949
(Showing 8 of 226 unconstrained eigenvalues)
```

# Adjusted R square

```
RsquareAdj(eth.rda_partial)
```

```
$r.squared
[1] 0.03279171
```

$adj.r.squared
[1] 0.009062113 # only 0.9% variance is explained

# Variance explained by RDA axes
The eigenvalues for the constrained axes reflect the variance explained by each canonical axis:

summary(eigenvals(eth.rda_partial, model = "constrained"))
Importance of components:

	RDA1	RDA2	RDA3	RDA4	RDA5	RDA6
Eigenvalue	8664.4153	7462.7486	7140.3382	5854.1430	5610.4140	4955.9774
Proportion Explained	0.2183	0.1880	0.1799	0.1475	0.1414	0.1249
Cumulative Proportion	0.2183	0.4063	0.5863	0.7338	0.8751	1.0000

# Visualize this information using a screeplot of the canonical eigenvalues.
jpeg('rda_partial_axes.jpg')
screeplot(eth.rda_partial)
dev.off()

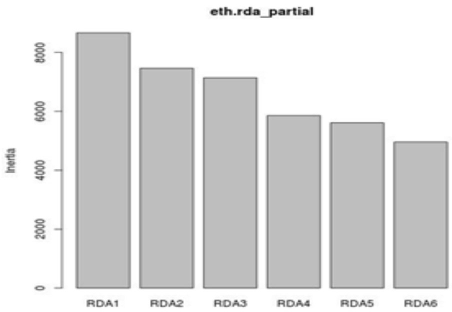

# Significance test for the RDA model
signif.partialModel_500perm <- anova.cca(eth.rda_partial, permutations=how(nperm=499), parallel=getOption("mc.cores"))

signif.partialModel_500perm

Permutation test for rda under reduced model
Permutation: free
Number of permutations: 499

Model: rda(formula = geno_onlyNumeric ~ minTemp + precSeasonality + precWQ + precDQ + SoilOrgC + LandUse + Condition(lat + long + k1 + k2 + k3), data = env, scale = T)

	Df	Variance	F	Pr(>F)	
Model	6	39688	1.3708	0.002	**
Residual	226	1090506			

Signif. codes: 0 '***' 0.001 '**' 0.01 '*' 0.05 '.' 0.1 ' ' 1

# Significance test for each axis

```
signif.axis <- anova.cca(eth.rda_partial,    permutations = how(nperm = 49), by="axis", parallel=getOption("mc.cores"))

signif.axis
```

```
Permutation test for rda under reduced model
Forward tests for axes
Permutation: free
Number of permutations: 49

Model: rda(formula = geno_onlyNumeric ~ minTemp + precSeasonality + precWQ + precDQ + SoilOrgC + LandUse + Condition(lat + long + k1 + k2 + k3), data = env, scale = T)
             Df Variance      F Pr(>F)
RDA1          1     8664 1.7956   0.02 *
RDA2          1     7463 1.5466   0.02 *
RDA3          1     7140 1.4798   0.02 *
RDA4          1     5854 1.2132   0.02 *
RDA5          1     5610 1.1627   0.02 *
RDA6          1     4956 1.0271   0.30
Residual 226   1090506
---
Signif. codes:   0 ‘***’ 0.001 ‘**’ 0.01 ‘*’ 0.05 ‘.’ 0.1 ‘ ’ 1
```

Identify outlier SNPs

SNP loadings in the ordination space will be used to determine outliers or candidates for local adaptation. The SNP loadings are stored as "species" in the RDA object. We’ll extract the SNP loadings from the significant constrained axes only. The SNPs that are in the two tails of SNP loading distribution will be considered as outliers.

# First check distribution of SNPs in all 5 significant axes.

```
loading.rda <- scores(eth.rda_partial, choices=c(1:5), display="species")
jpeg("SNP_loadings_histogram.jpg")
par(mfrow=c(3,2))
hist(loading.rda[,1], main="Loadings on RDA1")
hist(loading.rda[,2], main="Loadings on RDA2")
hist(loading.rda[,3], main="Loadings on RDA3")
hist(loading.rda[,4], main="Loadings on RDA4")
hist(loading.rda[,5], main="Loadings on RDA5")
dev.off()
```

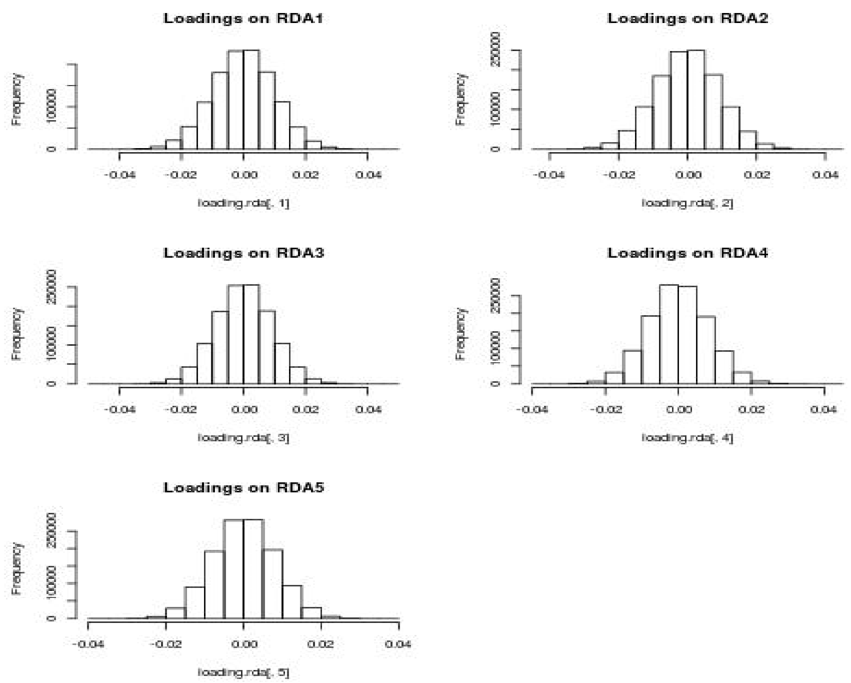

Given normal ditribution of SNP loading in all axes, SNPs that exceed SD>3.5 (two-tailed p-value = 0.0005) in both tails will be extracted as outliers using a function.

## Step1: Set a function to identify outliers:

```
outliers <- function(x,z){
+       lims <- mean(x) + c(-1, 1) * z * sd(x)
+       x[x < lims[1] | x > lims[2]]
+ }
```

# Step2: Now apply the function to identify the outlier snps from each significant RDA using SD=3.5

```
sd3.5.cand1 <- outliers(loading.rda[,1],3.5)
sd3.5.cand2 <- outliers(loading.rda[,2],3.5)
sd3.5.cand3 <- outliers(loading.rda[,3],3.5)
sd3.5.cand4 <- outliers(loading.rda[,4],3.5)
sd3.5.cand5 <- outliers(loading.rda[,5],3.5)
```

check the number of outliers in each axes

```
length(sd3.5.cand1)
[1] 523
length(sd3.5.cand2)
[1] 572
length(sd3.5.cand3)
[1] 662
length(sd3.5.cand4)
[1] 605
```

```
length(sd3.5.cand5)
[1] 504

# Total number of candidates from all axes
ncand.sd3.5 <- length(sd3.5.cand1) + length(sd3.5.cand2) + length(sd3.5.cand3) +     length(sd3.5.cand4) +
length(sd3.5.cand5)

ncand.sd3.5
[1] 2866
```

# Step 3: Organize the results by making one data frame with the axis, SNP name, loading, & correlation with each predictor.

```
sd3.5.cand1 <- cbind.data.frame(rep(1,times=length(sd3.5.cand1)), names(sd3.5.cand1), unname(sd3.5.cand1))
sd3.5.cand2 <- cbind.data.frame(rep(2,times=length(sd3.5.cand2)), names(sd3.5.cand2), unname(sd3.5.cand2))
sd3.5.cand3 <- cbind.data.frame(rep(3,times=length(sd3.5.cand3)), names(sd3.5.cand3), unname(sd3.5.cand3))
sd3.5.cand4 <- cbind.data.frame(rep(4,times=length(sd3.5.cand4)), names(sd3.5.cand4), unname(sd3.5.cand4))
sd3.5.cand5 <- cbind.data.frame(rep(5,times=length(sd3.5.cand5)), names(sd3.5.cand5), unname(sd3.5.cand5))

# Add column names as "axis", "snp" and "loading" to the above data frames
colnames(sd3.5.cand1) <- colnames(sd3.5.cand2) <- colnames(sd3.5.cand3) <- colnames(sd3.5.cand4) <-
colnames(sd3.5.cand5) <- c("axis","snp","loading")

# combine the outlier lists from all 5 axis
cand.5axes_sd3.5 <- rbind(sd3.5.cand1, sd3.5.cand2, sd3.5.cand3, sd3.5.cand4, sd3.5.cand5)
cand.5axes_sd3.5$snp <- as.character(cand.5axes_sd3.5$snp)

dim(cand.5axes_sd3)
[1] 2866      3

# Now get only the 6     predictor variables from the object "env" for downstream step
predictors <- subset (env, select=-c(long, lat, k1, k2, k3, Kebele))
predictors <- predictors[,2:7]                    # only selecting predictor columns, removing sample ID

str(predictors)
'data.frame':      238 obs. of     6 variables:
 $ minTemp              : int    73 74 74 71 72 74 72 74 52 52 ...
 $ precSeasonality: int    92 94 94 92 92 93 92 93 86 86 ...
 $ precWQ               : int    1002 1002 1000 1003 1003 1002 1001 999 1062 1062 ...
 $ precDQ               : int    48 45 45 50 48 46 48 45 92 92 ...
 $ SoilOrgC           : int    134 125 134 130 130 127 133 135 133 133 ...
 $ LandUse              : num    29.7 25.3 26.8 29 29.2 ...
```

# Add in the correlations of each outlier SNP with the 6 environmental predictors:

```
foo <- matrix(nrow=( ncand.sd3.5), ncol=6)   # 6 columns for 6 predictors
colnames(foo) <- c("minTemp","precSeasonality","precWQ","precDQ","SoilOrgC","LandUse")

for (i in 1:length(cand.5axes_sd3.5$snp)) {
+ nam <- cand.5axes_sd3.5[i,2]
+ snp.gen <- geno_onlyNumeric[,nam]
```

```
+ foo[i,] <- apply(predictors,2,function(x) cor(x,snp.gen))
+ }

cand.5axes_sd3.5 <- cbind.data.frame(cand.5axes_sd3.5,foo)

str(cand.5axes_sd3.5)

'data.frame':   2866 obs. of   9 variables:
 $ axis              : num  1 1 1 1 1 1 1 1 1 1 ...
 $ snp               : chr  "chr1_14897371" "chr1_23172610" "chr1_25140441" "chr1_25426203" ...
 $ loading           : num  0.0377 -0.0355 -0.0443 0.0383 -0.0372 ...
 $ minTemp           : num  -0.08931 0.00423 0.16819 0.03304 0.18331 ...
 $ precSeasonality: num  -0.163 -0.2072 -0.113 -0.0113 -0.1928 ...
 $ precWQ            : num  -0.1053 -0.1543 -0.2296 0.0551 -0.3305 ...
 $ precDQ            : num  0.092 0.2749 0.1887 -0.0925 0.1258 ...
 $ SoilOrgC          : num  0.0873 -0.3447 -0.3865 0.159 -0.2726 ...
 $ LandUse           : num  0.0917 0.0264 0.0285 -0.1002 0.1192 ...
```

#   Check and remove duplicated outlier snps
##   First, check number of duplicates among the outliers from all 5 axes

```
length(cand.5axes_sd3.5$snp[duplicated(cand.5axes_sd3.5$snp)])
[1] 3     # 3 duplicates
```

## Check which axis has duplicates

```
foo <- cbind(cand.5axes_sd3.5$axis, duplicated(cand.5axes_sd3.5$snp))

table(foo[foo[,1]==1,2])
  0
523

table(foo[foo[,1]==2,2])
  0
572

table(foo[foo[,1]==3,2])
    0    1
  661    1              # one duplicate in axis 3

table(foo[foo[,1]==4,2])
    0    1
  604    1              # one duplicate in axis 4

table(foo[foo[,1]==5,2])
    0    1
  503    1                  # one duplicate in axis 5
```

## Now remove duplicates

```
cand.5axes_sd3.5 <- cand.5axes_sd3.5[!duplicated(cand.5axes_sd3.5$snp),]
```

## Number of outliers after removing duplicates

```
dim(cand.5axes_sd3.5)
```

[1] 2863 9

# Find the most strongly correlated environmental predictor with each outlier SNP

```
for (i in 1:length(cand.5axes_sd3.5$snp)) {
bar <- cand.5axes_sd3.5[i,]
cand.5axes_sd3.5[i,10] <- names(which.max(abs(bar[4:9])))
cand.5axes_sd3.5[i,11] <- max(abs(bar[4:9]))}
colnames(cand.5axes_sd3)[10] <- "predictor"
colnames(cand.5axes_sd3)[11] <- "correlation"
```

In the above command, line3 gives the predictor name which has strongest correlation and save in column 10 in the cand.5axes_sd3.5 dataframe
Line4: gives the absolute value of correlation and save in column 11 in the cand.5axes_sd3.5 dataframe

# Tabulate the number of candidate SNPs for each predictor

```
table(cand.5axes_sd3.5$predictor)
```

LandUse	minTemp	precDQ	precSeasonality	precWQ	SoilOrgC
560	361	389	419	466	668

# Save the candidates in a output file for viewing over linux

```
write.table (cand.5axes_sd3.5, file="out02_candSNPs_pRDA_sd3.5_ancestryCorrected", quote=FALSE, sep ="\t", row.name =FALSE, col.name= TRUE)
```

<u>## Create RDA Plot</u>

Plot individuals in relation to RDA axes 1 and 2 with different color and symbols to different populations

```
population <-env$Kebele
pop_col <- as.integer(1:25)
pop_pch <- as.integer(1:25)
```

# RDA1 vs RDA2 without legend

```
jpeg("rda1_vs_rda2_ancestryCorrected.jpg")
plot(eth.rda_partial, choices = c(1, 2),    xlab="RDA1 (22%)", ylab="RDA2 (19%)", scaling=3)
points(eth.rda_partial, display="species", col="salmon",    scaling=3)
points(eth.rda_partial, display="sites", pch=pop_pch[population], cex=0.8, col=pop_col[population], scaling=3)
text(eth.rda_partial, scaling=3, display="bp", col="black", cex=1)
dev.off()
```

# Create only Legend and then crop legend part and place side by side the main plot

```
jpeg("pop_legend_ancestryCorrected.jpg")
par(xpd=T, mar=c(5,4,4,7))
plot(eth.rda_partial, type="n")
```

```
legend("topright", legend=levels(population), bty="o", col=pop_col, pch=pop_pch, cex=1)
dev.off()
```

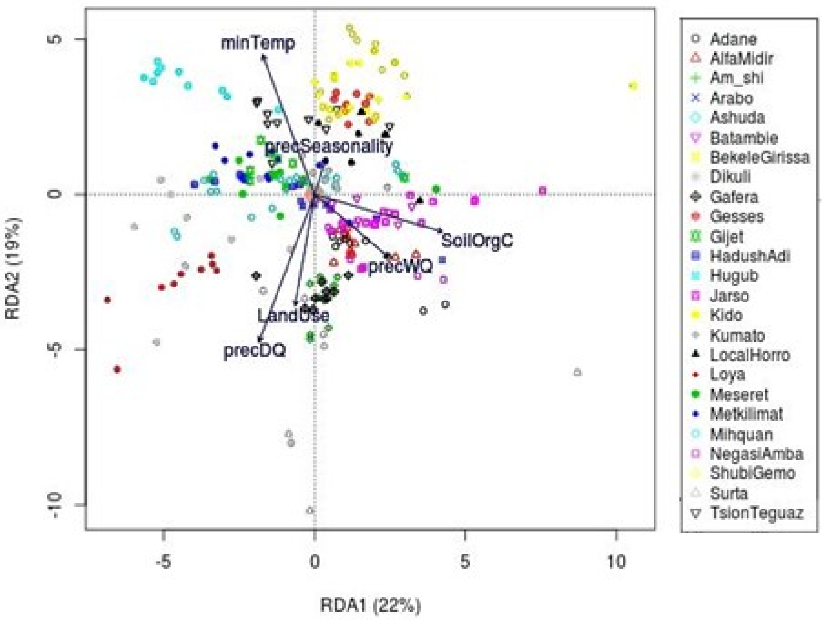

Supplement: msab156_Supplementary_Data [file msab156_supplementary_data.zip › SI_code_and_results_RDA.pdf]
