# Supplementary material for: Integrated Environmental and Genomic Analysis Reveals the Drivers of Local Adaptation in African Indigenous Chickens
Source: Mol Biol Evol. 2021 May 22;38(10):4268–85. doi: 10.1093/molbev/msab156 (PMC8476150; doi:10.1093/molbev/msab156)
Supplement: msab156_Supplementary_Data [file msab156_supplementary_data.zip › Supplementary_information.pdf]

**Supplementary Information**

# Integrated environmental and genomic analysis reveals the drivers of local adaptation in African indigenous chickens

Almas A. Gheyas, Adriana Vallejo-Trujillo, Adebabay Kebede, Maria Lozano-Jaramillo, Tadelle Dessie, Jacqueline Smith, Olivier Hanotte

Corresponding author: Almas A. Gheyas

Email: almas.gheyas@roslin.ed.ac.uk

**This file includes:**

- Supplementary methods
- Supplementary Figures: Figures S1 to S14
- Supplementary Tables: Tables S1 to S8 (Tables S3, S4, S5, and S7 are submitted in a separate supplementary excel file)
- Supplementary References

## Supplementary methods

### *Ecological Niche Modelling*

Environmental data for ecological modelling were downloaded from a number of public databases. Climatic data were extracted from the WorldClim database (v1 and v2) (Fick and Hijmans 2017). Soil property data were downloaded from the SoilGrids1km v0.5.8 database (Hengl, et al. 2014) and the Spatial Data Access Tool from NASA (ORNL_DAAC 2017). Data on land use and land cover were obtained from the Harmonized World Soil Database V1.2 (Fischer, et al. 2008). The Crop_dominance variable was accessed from the Global Food Security Analysis-Support Data (GFSAD30 2017).Supplementary Table S2 gives the full list of variables downloaded.

To avoid overfitting of the models, the environmental parameters were first shortlisted using the R package 'MaxentVariableSelection' (Jueterbock, et al. 2016) to remove correlated variables and select variables with large contribution. Model parameters were chosen by running MaxEnt with different combinations of Feature Classes (FCs: Linear, Quadratic, Product, Hinge, Categorical, and Threshold) and Regularisation Multiplier values (RM: 0.1 to 6 with steps of 0.5), and by evaluating the performance of these models based on AICc (**A**kaike **I**nformation **C**riterion **c**orrected for small samples) values with ENMeval (Muscarella, et al. 2014). The FC-RM combination with lowest AICc value was considered to be the best and was used for final models. Random*k*fold (with k = 10) method was used to partition "occurrence" data for training and testing purposes in ENMeval. The predictive power of the models was assessed using the Area Under ROC Curve (AUC) values by considering AUC > 0.5 as higher predictive power, AUC = 0.5 as random predictors, while values < 0.5 as worse than random predictor (Jimenez-Valverde 2012) (Supplementary Figure S6A). A jackknife assay was performed for the test and training data to assess the importance of the variables (Supplementary Figure S6B).

### *Mitigating the effects of population structure and demographic events on selection signature*

Hierarchical population structure and demographic history can bias results of selection signature through confounding effects. Since the Ethiopian populations investigated in this study showed low level of overall genetic differentiation (overall weighted *Fst* across all 25 population = 0.045; see Figure S2 for pairwise comparison of populations), we argue that population structure should not be a concern for the selection signature analyses performed in the study. Nevertheless, we still employed a number of mitigating measures to minimize any potential confounding effects of population structure and demographic events.

First, we deliberately combined two populations within each extreme group (Low and High) to reduce the potential impact of population structure. The rationale is that combining two populations within a group should create heterogeneity in the overall genome except in regions that are under selection, thereby improving the signal strength from adaptive regions while downgrading any false signal that may arise from population structure. The effectiveness of this approach can be observed in the Figure A below. The left hand panel of the Figure shows pairwise genome *Fst* among all populations used in each environmental analysis. As a general rule of thumb, *Fst* < 0.05 between populations would indicate little genetic differentiation, 0.05-0.15 as moderate genetic differentiation and > 0.15 as large differentiation (Hartl and Clark

1997; Balloux and Lugon-Moulin 2002). The Figure confirms that population differentiation is indeed quite low among our studied populations. For example, all pairs in precDQ, precSeason, soilOrgC and landUse analyses have *Fst* ≤ 0.05 and combining two populations per group reduces the *Fst* even further (see the Table at the bottom right in Figure A). Therefore, population structure issue should not be a concern in these cases. Only in MinTemp and precWQ analyses, we see a few cases of slightly higher *Fst* (with values 0.06, 0.08, 0.09 and 0.12). In these cases, combining two populations within Low and High groups also reduced the overall *Fst* between groups (minTemp=0.05 and precWQ=0.08).

| Fst | 0 | 0.1 | 0.2 | 0.3 | 0.4 | 0.5 | 0.6 | 0.7 | 0.8 | 0.9 | 1 |
|---|---|---|---|---|---|---|---|---|---|---|---|

**MinTemp**

| | AlfaMidir | NgAmba | Hugub | Mihquan |
|---|---|---|---|---|
| AlfaMidir | | | | |
| NgAmba | 0.01 | | | |
| Hugub | 0.09 | 0.09 | | |
| Mihquan | 0.06 | 0.05 | 0.08 | |

**precDQ**

| | Gijet | Kido | Kumato | Loya |
|---|---|---|---|---|
| Gijet | | | | |
| Kido | 0.03 | | | |
| Kumato | 0.03 | 0.04 | | |
| Loya | 0.04 | 0.05 | 0.01 | |

**precWQ**

| | Hugub | Jarso | Gafera | Gesses |
|---|---|---|---|---|
| Hugub | | | | |
| Jarso | 0.08 | | | |
| Gafera | 0.08 | 0.10 | | |
| Gesses | 0.10 | 0.12 | 0.02 | |

**precSeason**

| | Loya | Kumato | Meseret | Gijet |
|---|---|---|---|---|
| Loya | | | | |
| Kumato | 0.01 | | | |
| Meseret | 0.04 | 0.03 | | |
| Gijet | 0.04 | 0.03 | 0.01 | |

**soilOrgC**

| | Loya | Kumato | AlfaMidir | Adane |
|---|---|---|---|---|
| Loya | | | | |
| Kumato | 0.01 | | | |
| AlfaMidir | 0.05 | 0.05 | | |
| Adane | 0.03 | 0.03 | 0.01 | |

**landUse**

| | Gesses | Kido | Meseret | AlfaMidir |
|---|---|---|---|---|
| Gesses | | | | |
| Kido | 0.02 | | | |
| Meseret | 0.05 | 0.04 | | |
| AlfaMidir | 0.04 | 0.04 | 0.05 | |

Fst < 0.05 : little genetic differentiation
Fst 0.05 - 0.15 : moderate genetic differentiation
Fst 0.15 - 0.25 : large genetic differentiation
Fst > 0.25 : very large genetic differentiation

| | Low group | High group | Weighted Fst between groups | Fst values of sweep windows at ZFst >5 |
|---|---|---|---|---|
| minTemp | AlMidir, NgAmba | Hugub, Mihquan | 0.05 | 0.28-0.43 |
| precDQ | Gijet, Kido | Kumato, Loya | 0.03 | 0.21-0.42 |
| precWQ | Hugub, Jarso | Gafera, Gesses | 0.08 | 0.39-0.54 |
| Prec-Season | Loya, Kumato | Meseret, Gijet | 0.03 | 0.21-0.43 |
| soilOrgC | Loya, Kumato | AlMidir, Adane | 0.04 | 0.23-0.50 |
| landuse | Gesses, Kido | Meseret, AlMidir | 0.02 | 0.23-0.41 |

**Figure A:** Left hand panel shows pairwise weighted *Fst* values among populations used in different environmental analyses. Red and blue highlighted populations indicate Low and High groups respectively. Top right panel explains *Fst* value in relation to population differentiation. The Table at the bottom right shows the weighted *Fst* values between Low and High groups (after combining two populations) and *Fst* values in the detected sweep regions.

Apart from the approach above, we have employed very stringent criteria in identifying sweep regions. Instead of taking windows with only the top 1% of the sweep metrics, as is often practiced, we applied further stringent filtrations on standardised metrics (*zFst* > 5 and |XPEHH_std| >3). Using standardized values minimizes the effect of variations in population differentiation. For example using *zFst* > 5, changed the *Fst* thresholds of sweep regions depending on the level of population differentiation between groups (see the Table within the Figure A). Besides, the Table shows that the *Fst* values of the sweep regions are much higher - at least five times higher - compared to the overall *Fst* value between the groups, which should provide further confidence in our sweep calls.

Finally, we considered the common regions from XPEHH and *Fst* or extreme signals from individual methods (e.g. |XPEHH_std| > 4 or *zFst* > 8) as the strongest candidates. All these measures together are expected to provide a sufficiently robust method to avoid the confounding effects of population structure and demographic events.

## *XPEHH analysis*

XPEHH analyses were carried out using the Hapbin package (Maclean, et al. 2015). The genetic map positions for SNPs were calculated using chromosome-specific recombination rates based on (Groenen, et al. 2009) except for chr16 for which recombination rate was estimated based on Elferink *et al.* (Elferink, et al. 2010). Genotype data were phased in Beagle v5.1 (Browning and Browning 2007). XPEHH analyses were first performed for individual SNPs by setting options for minor allele frequency to be 5% and the cut-off value for Extended Haplotype Homozygosity (EHH) to be 0.1. Subsequently, mean values were calculated within windows. Only windows with all the SNPs showing the same directionality of selection were considered for sweep detection, with positive XPEHH values indicating selection in the Low group, and negative values representing selection in the High group.

## *Redundancy Analysis*

RDA was performed in Vegan v2.5-4 in R (Oksanen 2015) following (Forester 2019). Autosomal SNPs - without any missing genotypes - were first LD pruned using PLINK v1.9 with the “--indep-pairwise” command with window size = 10kb, step size = 10 SNPs and $r^2 > 0.5$. The genotype data of the remaining sites were converted as dosage (number) of non-reference allele (0, 1 and 2). RDA was run with genotypes as the response variables and environmental data as the explanatory variables, conditioned on latitude, longitude and ancestry-coefficients for three ancestral gene pools ($K$=3) from ADMIXTURE analysis. Significance of the overall model and the RDA axes were estimated by the anova.cca function in Vegan with permutation numbers of 499 and 49, respectively. Given the normal distribution of SNP loadings, a cut-off value of SD >= 3.5 was first used for detecting outliers (two-tailed $P$-value = 0.0005) for each significant axis. Then further filtration was applied to retain only those SNPs as candidates that showed environmental correlation ≥ 0.3. SNPs in complete LD ($r^2 = 0.1$) with the candidates were identified using PLINK by specifying the following options:

*--r2 --ld-snp-list <candidate_SNP_list> --ld-window-r2 1 --ld-window-kb 40kbs.*

## Supplementary Figures

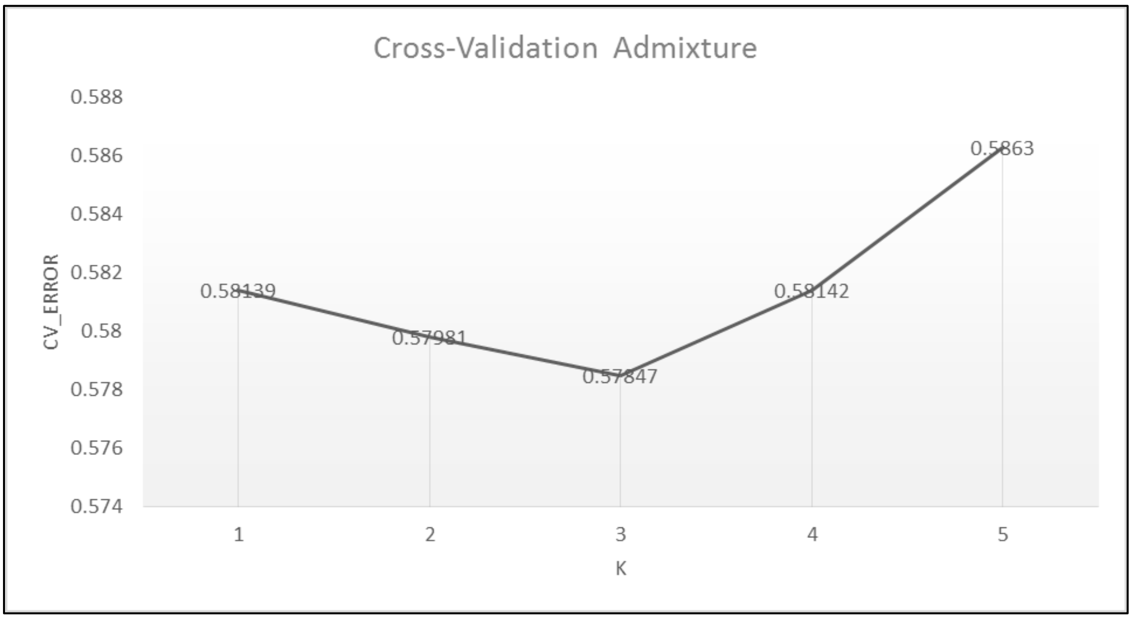


**Figure S1.** Cross-validation showing K=3 as the best value from Admixture analysis explaining ancestry for the 25 Ethiopian chicken population investigated.

| Fst | 0 | 0.1 | 0.2 | 0.3 | 0.4 | 0.5 | 0.6 | 0.7 | 0.8 | 0.9 | 1 |
|---|---|---|---|---|---|---|---|---|---|---|---|

| | Adane | AlfaMidir | AmShi | Arabo | Ashuda | Batambie | BekeleGirissa | Dikuli | Gafera | Gesses | Gijet | HadushAdi | Hugub | Jarso | Kido | Kumato | Horro | Loya | Meseret | Metkilmat | Mihquan | NegasiAmba | ShubiGemo | Surta | TsionTeguaz |
|---|---|---|---|---|---|---|---|---|---|---|---|---|---|---|---|---|---|---|---|---|---|---|---|---|---|
| Adane | | | | | | | | | | | | | | | | | | | | | | | | | |
| AlfaMidir | 0.01 | | | | | | | | | | | | | | | | | | | | | | | | |
| AmShi | 0.02 | 0.04 | | | | | | | | | | | | | | | | | | | | | | | |
| Arabo | 0.00 | 0.01 | 0.02 | | | | | | | | | | | | | | | | | | | | | | |
| Ashuda | 0.03 | 0.05 | 0.01 | 0.03 | | | | | | | | | | | | | | | | | | | | | |
| Batambie | 0.02 | 0.05 | 0.01 | 0.03 | 0.02 | | | | | | | | | | | | | | | | | | | | |
| BekeleGirissa | 0.03 | 0.05 | 0.03 | 0.04 | 0.04 | 0.03 | | | | | | | | | | | | | | | | | | | |
| Dikuli | 0.02 | 0.04 | 0.00 | 0.02 | 0.01 | 0.01 | 0.03 | | | | | | | | | | | | | | | | | | |
| Gafera | 0.02 | 0.04 | 0.00 | 0.02 | 0.01 | 0.01 | 0.03 | 0.00 | | | | | | | | | | | | | | | | | |
| Gesses | 0.03 | 0.04 | 0.02 | 0.03 | 0.03 | 0.03 | 0.06 | 0.02 | 0.02 | | | | | | | | | | | | | | | | |
| Gijet | 0.02 | 0.05 | 0.02 | 0.03 | 0.03 | 0.02 | 0.03 | 0.02 | 0.02 | 0.05 | | | | | | | | | | | | | | | |
| HadushAdi | 0.04 | 0.08 | 0.04 | 0.06 | 0.05 | 0.04 | 0.04 | 0.04 | 0.04 | 0.07 | 0.03 | | | | | | | | | | | | | | |
| Hugub | 0.08 | 0.09 | 0.08 | 0.08 | 0.09 | 0.09 | 0.07 | 0.08 | 0.08 | 0.10 | 0.09 | 0.10 | | | | | | | | | | | | | |
| Jarso | 0.09 | 0.11 | 0.10 | 0.10 | 0.11 | 0.11 | 0.09 | 0.10 | 0.10 | 0.12 | 0.10 | 0.12 | 0.08 | | | | | | | | | | | | |
| Kido | 0.03 | 0.04 | 0.02 | 0.03 | 0.03 | 0.02 | 0.04 | 0.02 | 0.02 | 0.02 | 0.03 | 0.06 | 0.10 | 0.11 | | | | | | | | | | | |
| Kumato | 0.03 | 0.05 | 0.03 | 0.04 | 0.04 | 0.03 | 0.02 | 0.03 | 0.03 | 0.05 | 0.03 | 0.05 | 0.07 | 0.09 | 0.04 | | | | | | | | | | |
| Horro | 0.02 | 0.05 | 0.01 | 0.03 | 0.03 | 0.02 | 0.03 | 0.01 | 0.01 | 0.03 | 0.02 | 0.04 | 0.09 | 0.11 | 0.02 | 0.03 | | | | | | | | | |
| Loya | 0.03 | 0.05 | 0.03 | 0.04 | 0.05 | 0.04 | 0.02 | 0.03 | 0.04 | 0.06 | 0.04 | 0.05 | 0.08 | 0.09 | 0.05 | 0.01 | 0.03 | | | | | | | | |
| Meseret | 0.02 | 0.05 | 0.02 | 0.03 | 0.03 | 0.02 | 0.03 | 0.02 | 0.02 | 0.05 | 0.01 | 0.03 | 0.08 | 0.10 | 0.04 | 0.03 | 0.02 | 0.04 | | | | | | | |
| Metkilmat | 0.02 | 0.04 | 0.02 | 0.03 | 0.03 | 0.02 | 0.03 | 0.02 | 0.02 | 0.04 | 0.00 | 0.03 | 0.08 | 0.10 | 0.03 | 0.03 | 0.02 | 0.04 | 0.00 | | | | | | |
| Mihquan | 0.03 | 0.06 | 0.02 | 0.04 | 0.04 | 0.03 | 0.03 | 0.02 | 0.03 | 0.05 | 0.01 | 0.02 | 0.08 | 0.10 | 0.04 | 0.03 | 0.03 | 0.04 | 0.01 | 0.01 | | | | | |
| NegasiAmba | 0.01 | 0.01 | 0.03 | 0.01 | 0.04 | 0.04 | 0.04 | 0.03 | 0.03 | 0.04 | 0.05 | 0.07 | 0.09 | 0.11 | 0.04 | 0.04 | 0.04 | 0.05 | 0.04 | 0.04 | 0.05 | | | | |
| ShubiGemo | 0.03 | 0.06 | 0.04 | 0.04 | 0.05 | 0.04 | 0.02 | 0.04 | 0.04 | 0.07 | 0.03 | 0.05 | 0.08 | 0.10 | 0.05 | 0.03 | 0.04 | 0.03 | 0.03 | 0.03 | 0.04 | 0.05 | | | |
| Surta | 0.02 | 0.05 | 0.01 | 0.03 | 0.02 | 0.01 | 0.02 | 0.00 | 0.01 | 0.04 | 0.02 | 0.04 | 0.08 | 0.10 | 0.03 | 0.02 | 0.02 | 0.03 | 0.02 | 0.02 | 0.02 | 0.04 | 0.03 | | |
| TsionTeguaz | 0.01 | 0.03 | 0.01 | 0.02 | 0.02 | 0.02 | 0.03 | 0.01 | 0.01 | 0.02 | 0.02 | 0.04 | 0.09 | 0.10 | 0.02 | 0.03 | 0.02 | 0.04 | 0.02 | 0.01 | 0.03 | 0.03 | 0.04 | 0.02 | |

**Figure S2:** Heat map of genomic weighted *Fst* of pairwise populations. Total number of population is 25 with 300 pairwise comparisons.

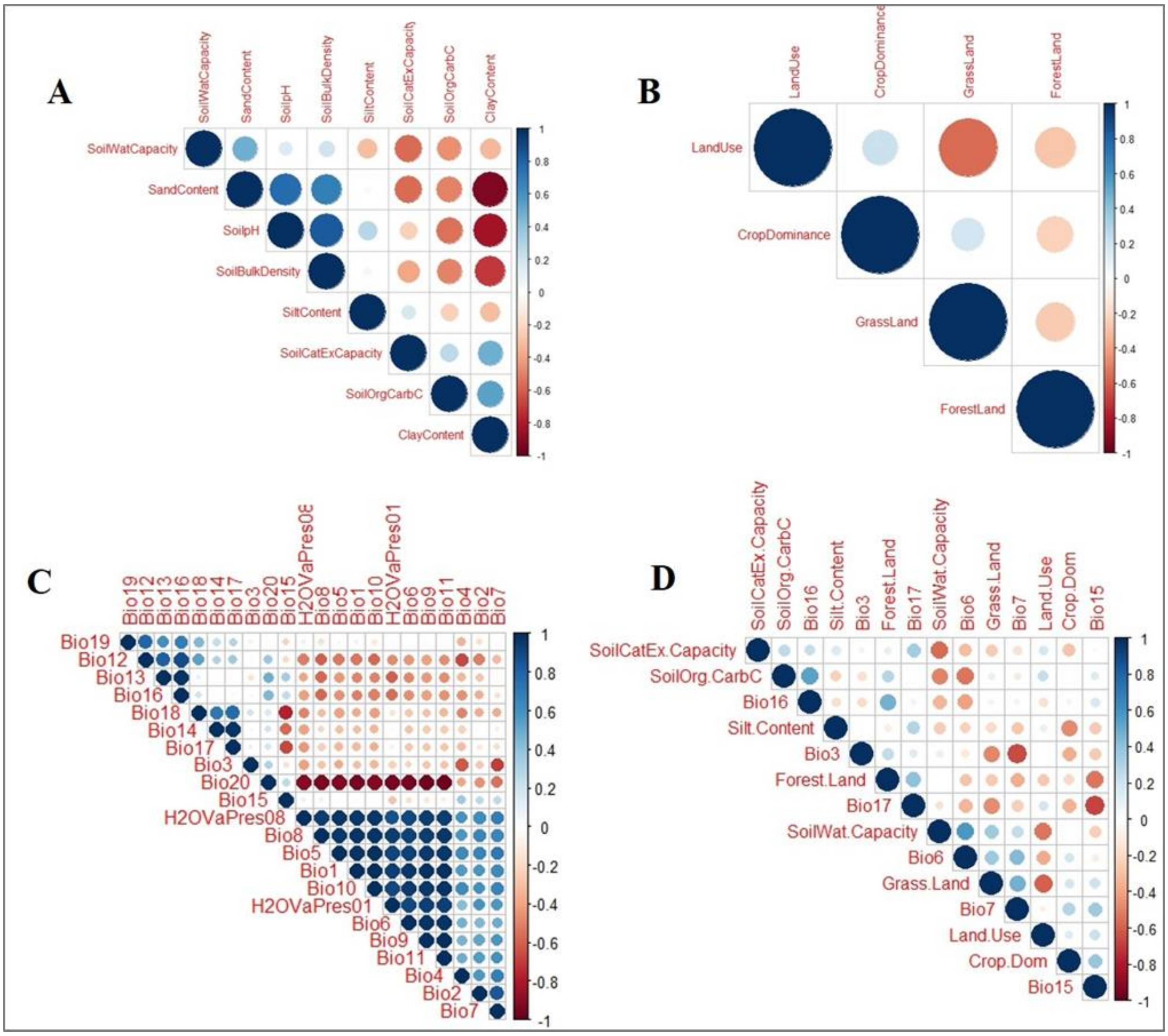


**Figure S3.** Spearman correlation analysis of environmental parameters: (A) Soil variables, (B) Vegetation and land cover variables, (C) Climatic variables, (D) correlation analysis between non-correlated variables from different groups.

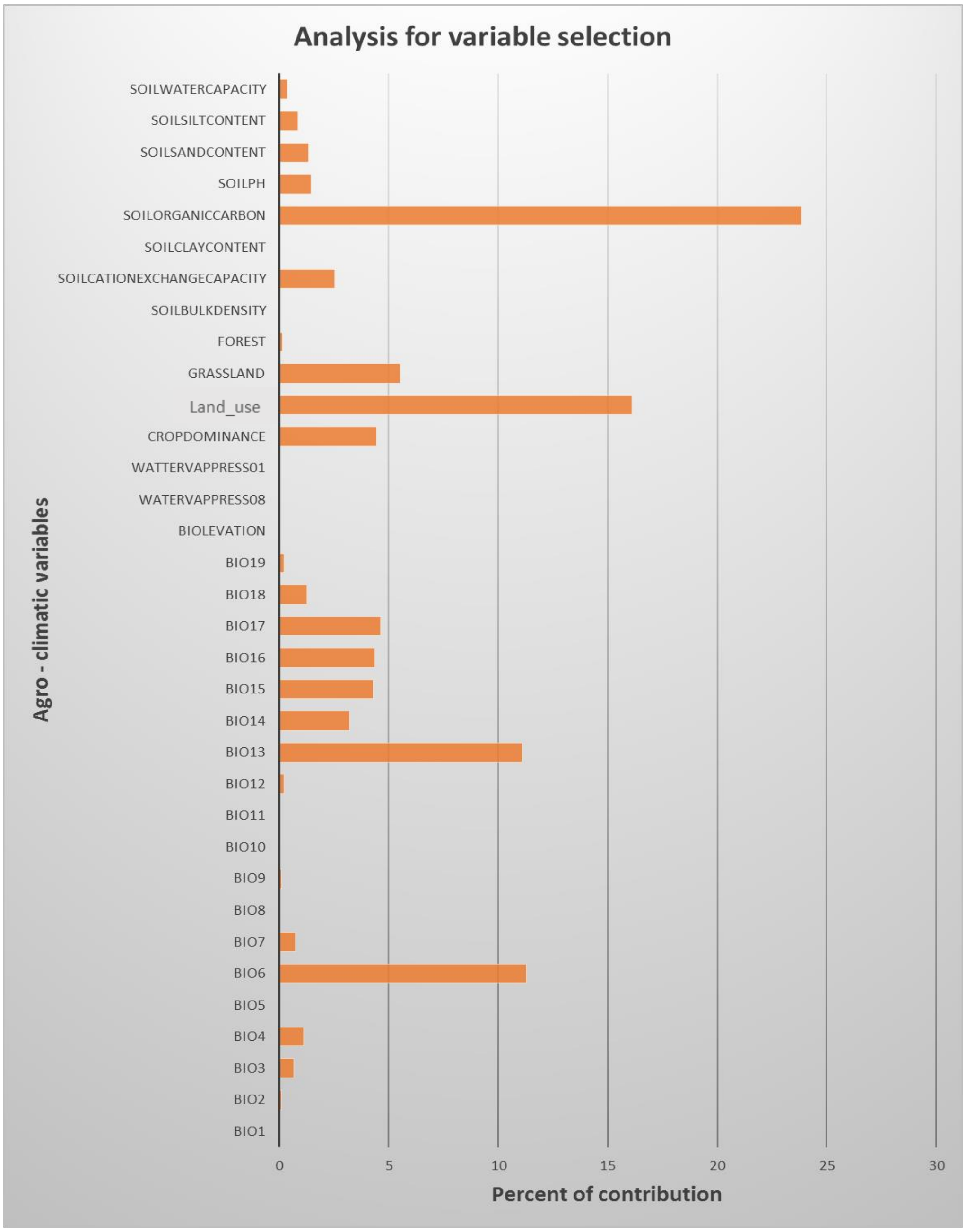


**Figure S4.** Percentage contributions of each of the 34 agro-climatic variables calculated using the MaxEntVariableSelection R package. Variables with values >= 4% were retained for the final model

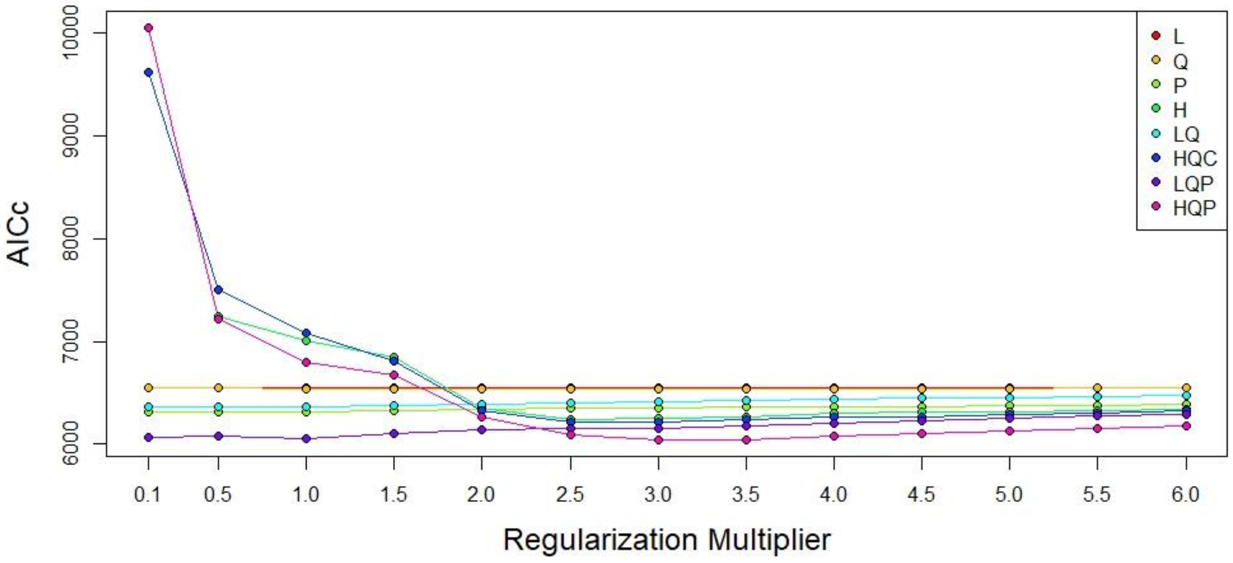


**Figure S5:** AICc values for different combinations of Feature Classes (FCs) and Regularization Multipliers. The FCs shown are: Linear (L), Quadratic (Q), Product (P), Hinge (H) and Categorical (C). The Threshold FC is not depicted here, as it did not show any effect in any combination.

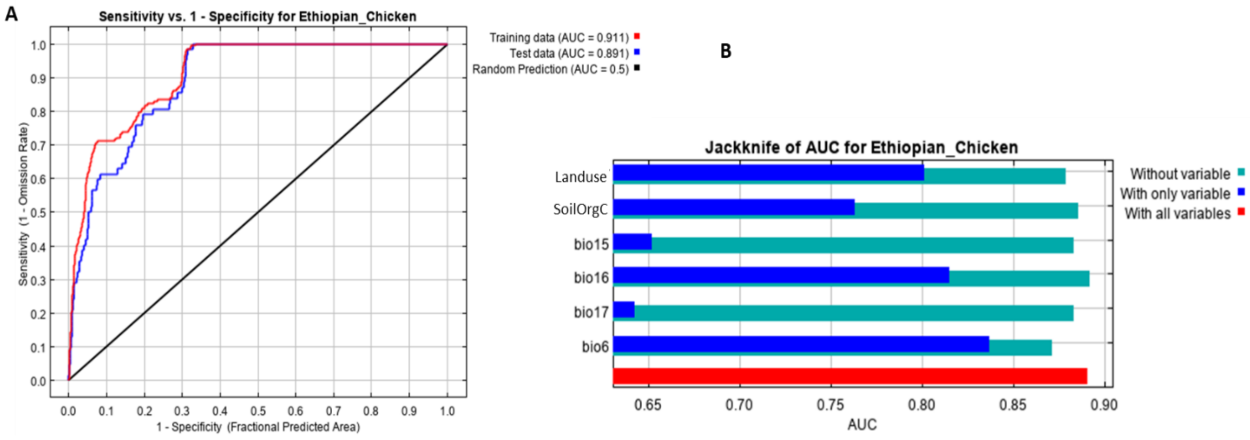


**Figure S6.** MaxEnt model based on the six selected variables (A) Receiver Operating Curve for training and test data, (B) Jackknife result for AUC (Area Under Receiver Operating Curve) for each selected environmental predictors.

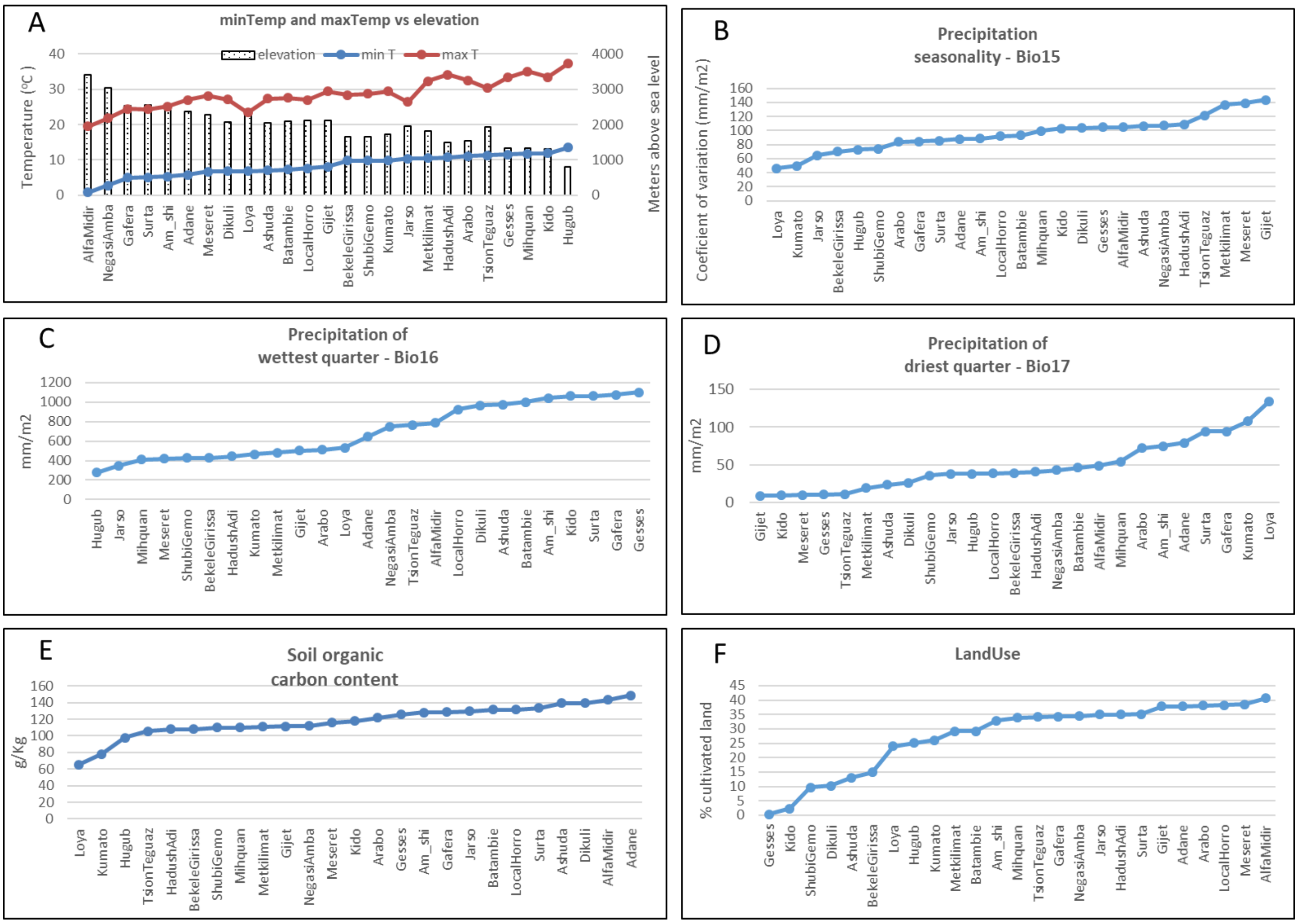


**Figure S7:** Population gradients based on the six key environmental parameters used for Ecological Niche Modelling. For each environmental parameter, two populations from each end of the distribution were chosen for selection signature analysis. Figure S5A shows the minimum temperature in the coldest month (minTemp; Bio6) along with the maximum temperature of the warmest month (Bio5) and elevation (Bio20) due to their strong correlation. For the temperature/elevation High group, Mihquan instead of Kido was chosen, as in Mihquan temperature parameters in both the coldest and warmest months were among the highest and elevation among the lowest compared to other populations.

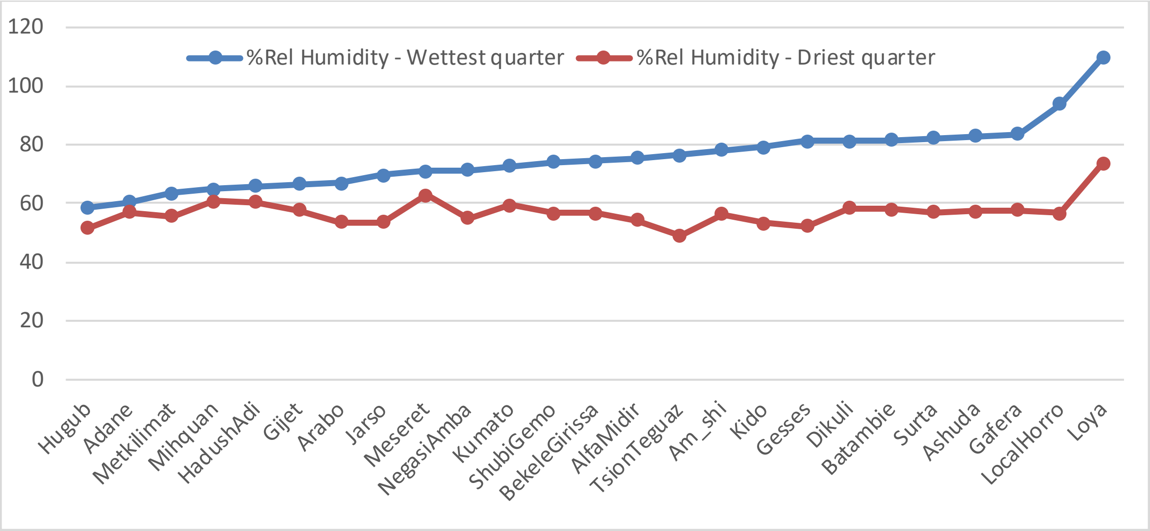


**Figure S8:** Mean percentage relative humidity in different populations in the wettest and driest quarter

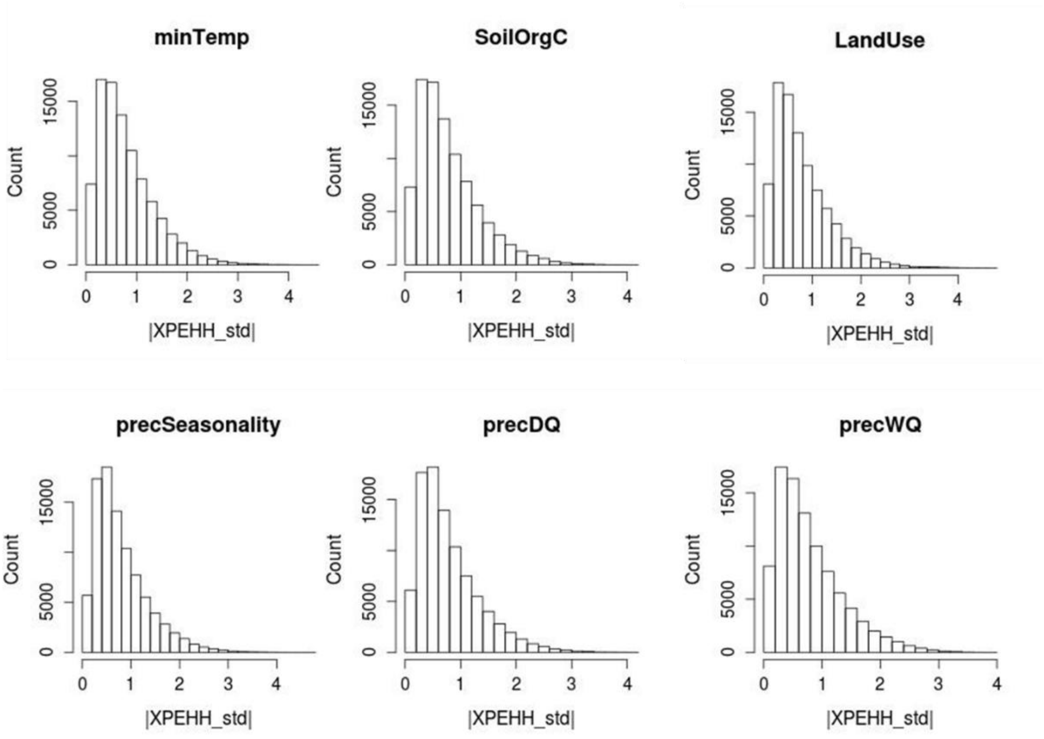


**Figure S9:** Histograms of |XPEHH| values in windows in different analyses

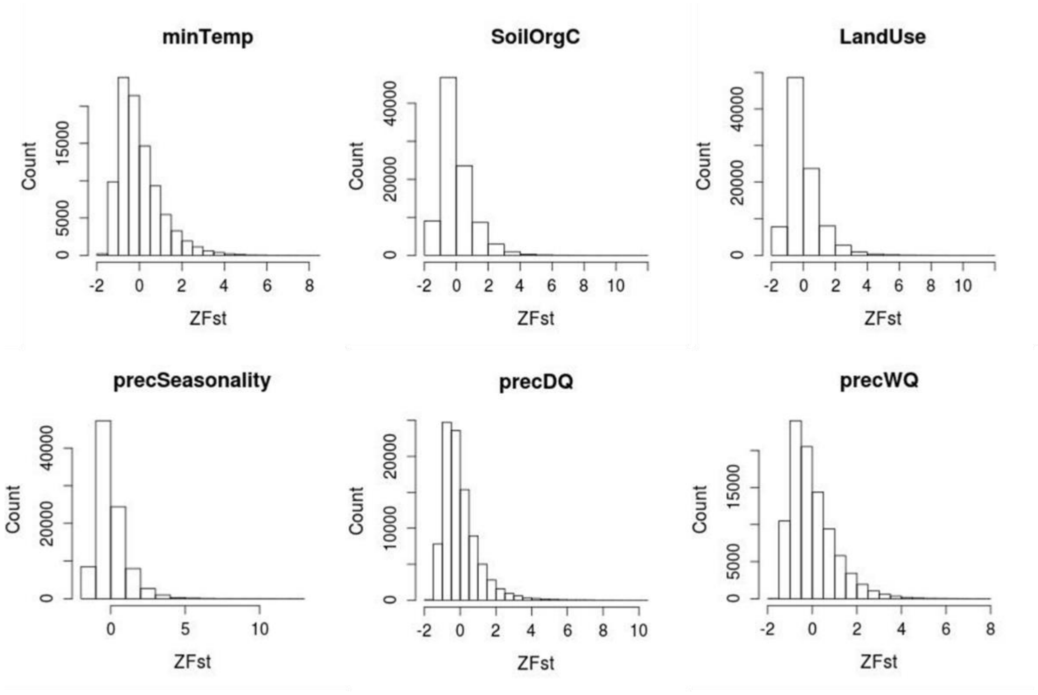


**Figure S10:** Histograms of *ZFst* values in windows in different analyses

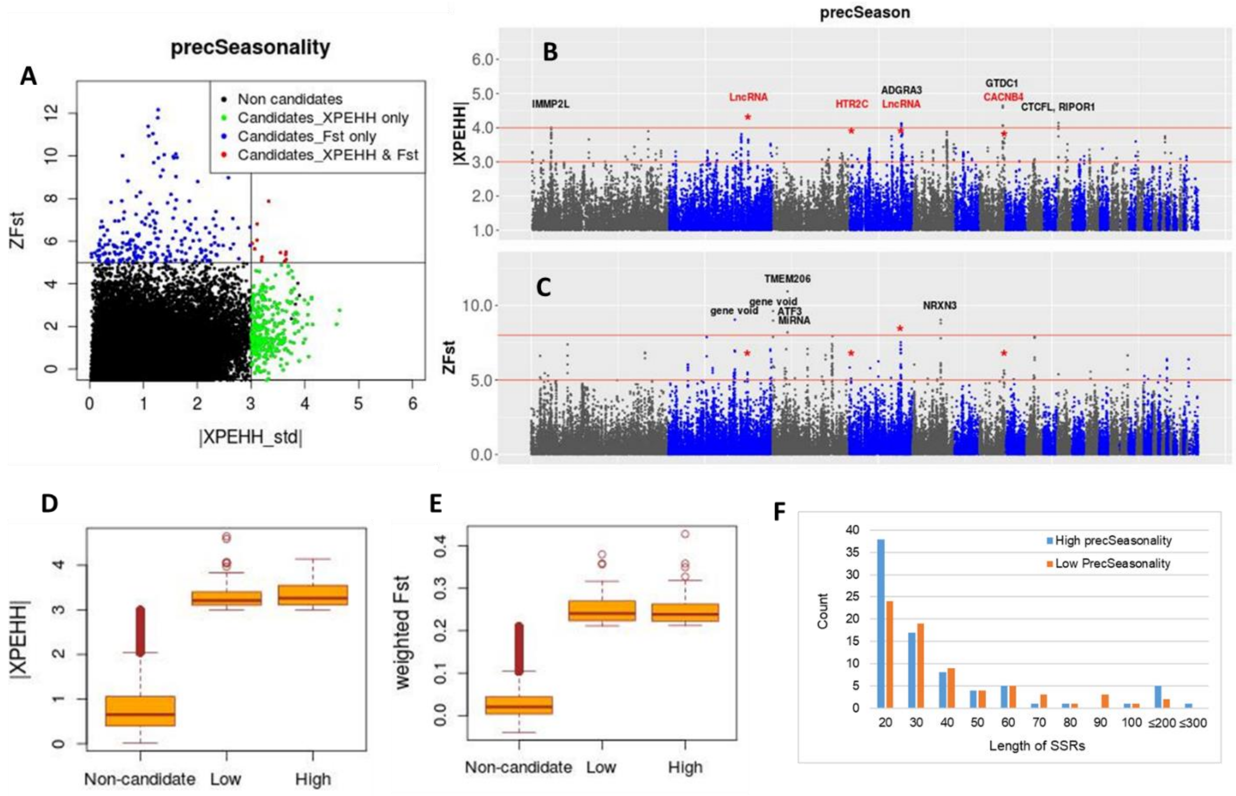


**Figure S11:** Selection signature analysis results for precSeasonality. (A) Scatter plot of standardaized values of XPEHH vs *Fst*. (B-C) Manhattan plots for the XPEHH and *Fst* analyses; common windows are marked with asterisk and gene names from common windows are shown in red. (D-E) Box plots showing the distribution of *Fst* and XPEHH metrics for non-candidate and candidate windows from Low and High groups. (F) Length distribution of selective Sweep Regions (SRs).

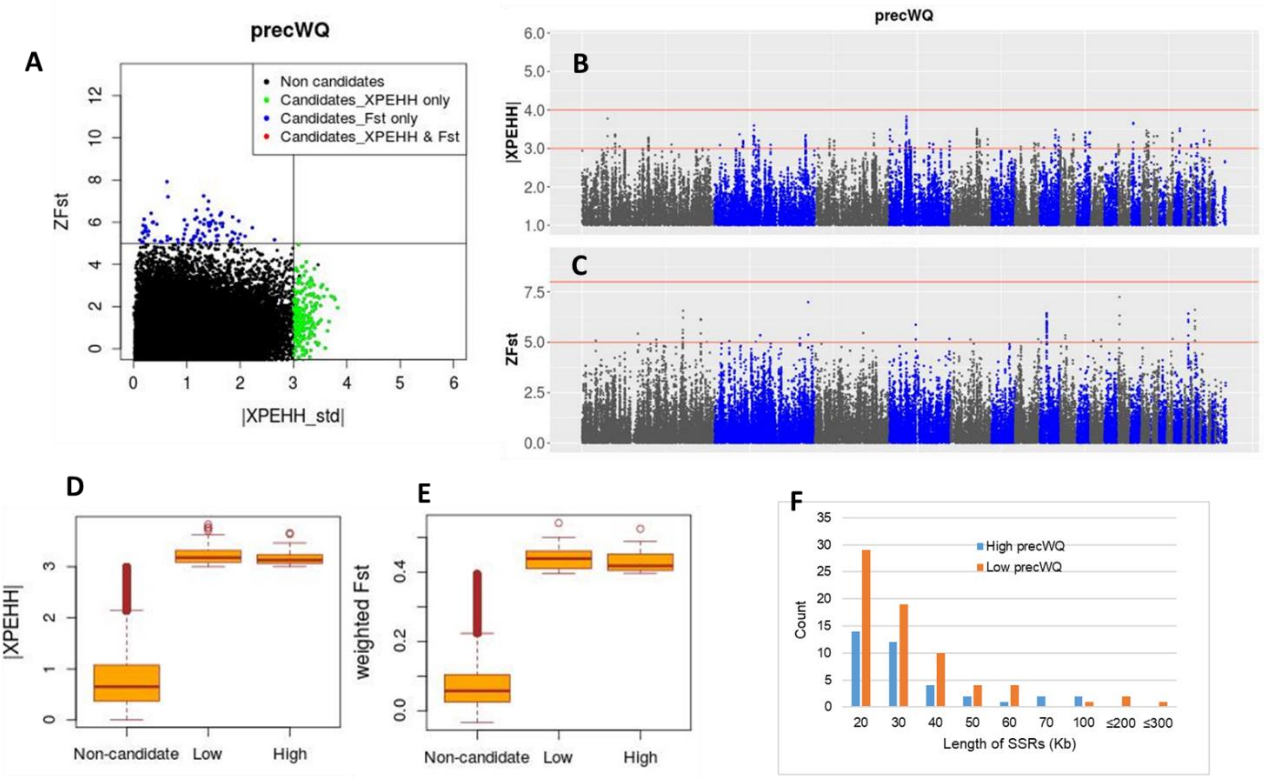


**Figure S12:** Selection signature analysis results for precWQ. (A) Scatter plot of standardaized values of XPEHH vs *Fst*. (B-C) Manhattan plots for the XPEHH and *Fst* analyses. (D-E) Box plots showing the distribution of *Fst* and XPEHH metrics for non-candidate and candidate windows from Low and High groups. (F) Length distribution of selective Sweep Regions (SRs).

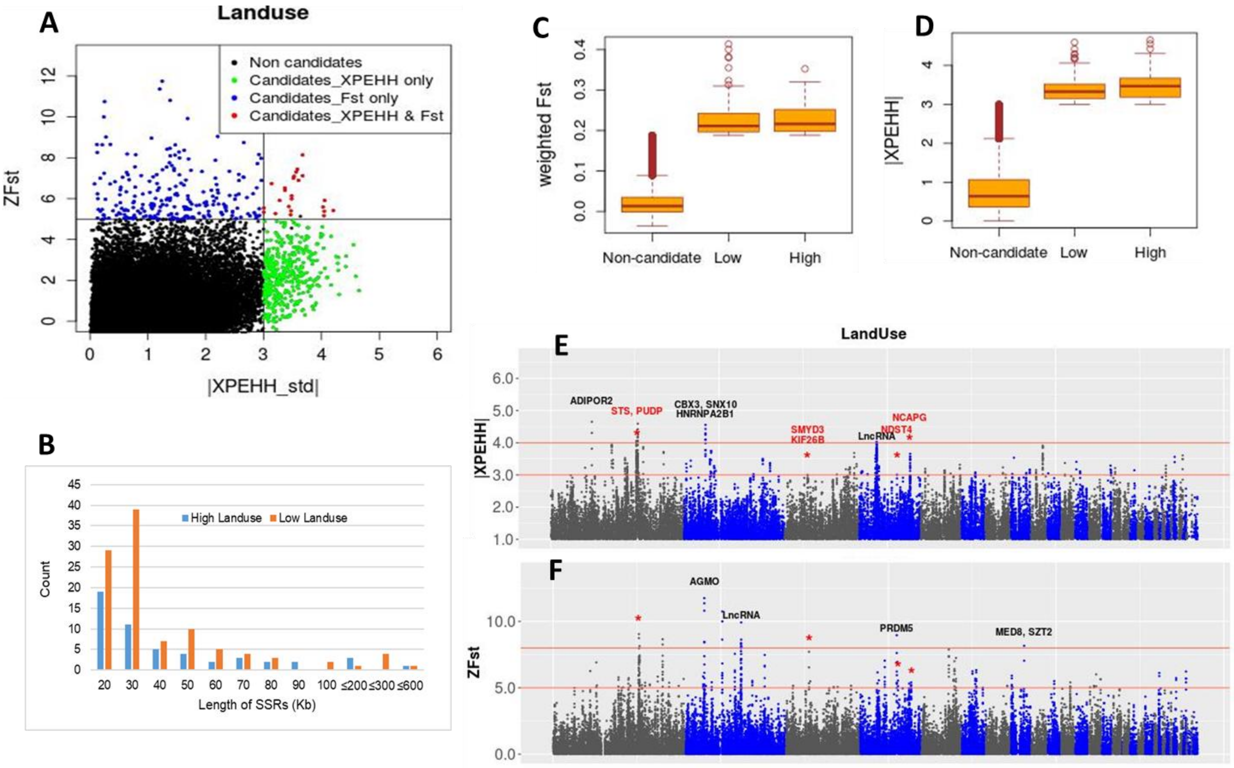


**Figure S13:** Selection signature analysis results for Landuse. (A) Scatter plot of standardaized values of XPEHH vs *Fst*. (B) Length distribution of selective Sweep Regions (SRs). (C-D) Box plots showing the distribution of *Fst* and XPEHH metrics for non-candidate windows and the candidate windows for Low and High groups. (E-F) Manhattan plots for the XPEHH and *Fst* analyses; common windows are marked with asterisk and gene names from common windows are shown in red.

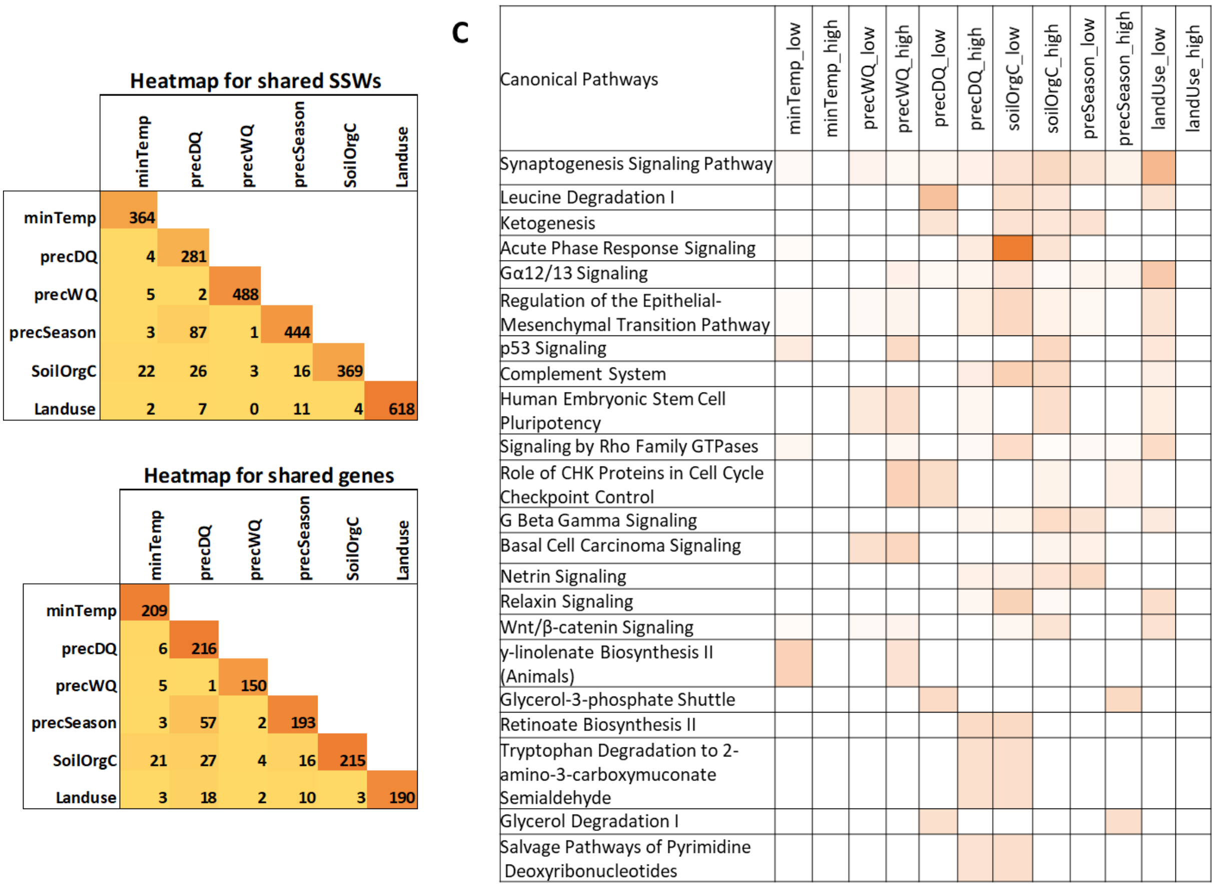


**Figure S14:** Shared selective sweep candidates among different environmental analysis. (A) Shared candidate windows; (B) shared candidate genes; (C) shared canonical pathways affected by candidate genes from different environmental analyses. Only pathways showing –log(*P*-value) ≥ 2 in at least two environmental analyses are shown.

## Supplementary Tables

### Table S1. Ethiopian chicken populations studied along with the characteristics and classification of their geographic locations.

| Population | N | Agro-ecological Zone (AEZ-16) | Traditional classification | Geographic Region | District | Elevation | Slope |
|---|---|---|---|---|---|---|---|
| Adane | 10 | Tropic – cool/semiarid, sub-humid | Dega | Amhara | Kalu | 2280 - 2734 | East |
| Alfa Midir | 10 | Tropic – cool/sub-humid | Dega | Amhara | Menz Gera Midir | 3304 - 3451 | East |
| Amesha Shinkuri | 10 | Tropic – cool/sub-humid | Dega | Amhara | Fagita Lekoma | 2417 - 2564 | West |
| Arabo | 10 | Tropic – cool/semiarid | Weina - dega | Amhara | Kalu | 1624 - 1471 | East |
| Ashuda | 10 | Tropic – cool/sub-humid | Weina - dega | Amhara | South Achefer | 2009 - 2103 | West |
| Batambie | 8 | Tropic – cool/sub-humid | Weina - dega | Amhara | Banja | 2003 - 2127 | West |
| Bekele Girissa | 10 | Tropic – cool/sub-humid | Weina - dega | Oromia | Dugda | 1642 - 1668 | Center |
| Dikuli | 10 | Tropic – cool/sub-humid | Weina - dega | Amhara | South Achefer | 2022 - 2109 | West |
| Gafera | 10 | Tropic – cool/sub-humid | Dega | Amhara | Fagita Lekoma | 2441 - 2592 | West |
| Gesses | 9 | Tropic – warm/humid | Kolla | Gumuz | Dibate | 1196 - 1546 | West |
| Gijet | 9 | Tropic – cool/semiarid | Weina - dega | Tigray | Sahareti Samire | 1990 - 2303 | East |
| Hugub | 10 | Tropic – warm/semiarid | Kolla | Afar | Dulecha | 737 - 979 | East |
| Hadush Adi | 9 | Tropic – cool/semiarid | Kolla | Tigray | Merebleke | 1417 - 1669 | East |
| Jarso | 14 | Tropic – cool/semiarid | Weina - dega | Oromia | Jarso | 1817 - 2136 | East |
| Kido | 9 | Tropic – cool/ sub-humid | Kolla | Gumuz | Dibate | 1281 - 1418 | West |
| Kumato | 10 | Tropic – cool/sub-humid | Weina - dega | SNNPR | Dara | 1838 - 1969 | East |
| Local Horro | 6 | Tropic – cool/sub-humid | Weina - dega | Oromia | Horro | 1921 - 2375 | West |
| Loya | 10 | Tropic – cool/sub-humid | Weina - dega | SNNPR | Dara | 1601 - 1847 | East |
| Meseret | 10 | Tropic – cool/semiarid | Weina - dega | Tigray | Enderta | 2248 - 2312 | East |
| Metkilimat | 10 | Tropic – cool/semiarid | Weina - dega | Tigray | Sahareti Samire | 1709 - 2051 | East |
| Mihquan | 10 | Tropic – cool/semiarid | Kolla | Tigray | Merebleke | 1295 - 1495 | East |
| Negasi Amba | 10 | Tropic – cool/sub-humid | Dega | Amhara | Menz Gera Midir | 2978 - 3071 | East |
| Shubi Gemo | 10 | Tropic – cool/sub-humid | Weina - dega | Oromia | Dugda | 1643 - 1651 | Center |
| Surta | 9 | Tropic – cool/sub-humid | Dega | Amhara | Banja | 2480 - 2575 | West |
| Tsion Teguaz | 10 | Tropic – cool/semiarid | Weina - dega | Amhara | Gondar Zuria | 1870 - 1968 | West |

*SNNPR: Southern Nations, Nationalities, and People's Region)

## Table S2. Climatic and agro-ecological variables tested in Ecological Niche Modelling for Ethiopian chicken

| | Variable | Description | Units | Database |
|---|---|---|---|---|
| Climatic variables | Bio1 | Annual Mean Temperature | °C | WorldClim - Global climate data http://worldclim.org |
| | Bio2 | Mean Diurnal Range | °C (Bio2/Bio7) | |
| | Bio3 | Isothermality | °C | |
| | Bio4 | Temperature Seasonality | °C | |
| | Bio5 | Max temperature of warmest month | °C | |
| | Bio6 | Min temperature of coldest month | °C | |
| | Bio7 | Temperature annual range | °C (Bio5 - Bio6) | |
| | Bio8 | Mean temperature of wettest quarter | °C | |
| | Bio9 | Mean temperature of driest quarter | °C | |
| | Bio10 | Mean temperature of warmest quarter | °C | |
| | Bio11 | Mean temperature of coldest quarter | °C | |
| | Bio12 | Annual precipitation | mm/m2 | |
| | Bio13 | Precipitation of wettest month | mm/m2 | |
| | Bio14 | precipitation of driest month | mm/m2 | |
| | Bio15 | Precipitation seasonality | mm/m2 (Coeficient of variation) | |
| | Bio16 | Precipitation of wettest quarter | mm/m2 | |
| | Bio17 | Precipitation of driest quarter | mm/m2 | |
| | Bio18 | Precipitation of warmest quarter | mm/m2 | |
| | Bio19 | Precipitation of coldest quarter | mm/m2 | |
| | WatVapPress01 | Water vapour pressure of the wettest month | kPa | |
| | WatVapPress08 | Water vapour pressure of the driest month | kPa | |
| | Elevation | Meters above sea level | m.a.s.l. | |
| Soil variables | soil_pH | Soil pH | pH (x10 in H2O) | Global gridded soil information https://soilgrids.org |
| | CatEx_Capacity | Cation Exchange capacity | cmolc/Kg at depht 0.00 m | |
| | BulkD | Bulk Density | Kg/m3 at depht 0.00 m | |
| | Organic_Carbon | Soil organic carbon content | g/Kg at depht 0.00 m | |
| | Clay | Clay content | mass fraction in % at depht 0.00 m | |
| | Silt | Silt content | mass fraction in % at depht 0.00 m | |
| | Sand | Sand content | mass fraction in % at depht 0.00 m | |
| | WaterCapacity | Total available water capacity | mm2/1mt soil depht | Spatial Data Access Tool (SDAT) - NASA https://webmap.ornl.gov/ogc/wcsdown.jsp?dg_id= |
| Vegetation variables | Forest | Forest cover | % | Harmonized World Soil Dataset http://www.fao.org/soils-portal/soil-survey/soil-maps-and-databases/harmonized-world-soil-database-v12/en/ |
| | Grassland | Grass/shrub cover | % | |
| | Landuse | Land use for agricultural purposes | % | |
| | Crop_dominance | Crop dominance (majors crops) | Category | Global Food S3ecurity Analysis-Support DATA https://www.croplands.org/ |

**Table S3: Candidate selection signature regions and overlapping genes detected from analyses of different environmental parameters.**

See worksheet: “TableS3_SRs_overlapping_genes” under the supplementary file “SI_Tables_S3_S4_S5_S7.xlsx”

**Table S4: Candidate SNP list from selection signature analysis**

See worksheet: “TableS4_candSNPs_SSA” under the supplementary file “SI_Tables_S3_S4_S5_S7.xlsx”

**Table S5: Overlap of QTLs with candidate genes from selection signature regions.**

See worksheet: “TableS5_SSA_overlappingQTLs” under the supplementary file “SI_Tables_S3_S4_S5_S7.xlsx”

**Table S6A: Top molecular and cellular functions and physiological processes affected by candidate genes involved in adaptation to temperature and altitude**

| **Low temperature/High altitude** | **High temperature/Low altitude** |
| --- | --- |
| **Top molecular and cellular function**<br>- Lipid Metabolism<br>- Small Molecule Biochemistry<br>- Molecular Transport<br>- Cellular Assembly and Organization<br>- Cell-to-cell Signalling and Interactions | **Top molecular and cellular function**<br>- Cell Cycle<br>- Cell-To-Cell Signalling and Interaction<br>- Cellular Development<br>- Cellular Growth and Proliferation<br>- Cellular Movement |
| **Physiological system development and function**<br>- Renal and Urological System Development and Function<br>- Haematological System Development and Function<br>- Tissue Development<br>- Connective Tissue Development and Function<br>- Embryonic Development | **Physiological system development and function**<br>- Reproductive System Development and Function<br>- Nervous System Development and Function<br>- Cardiovascular System Development and Function<br>- Organ Morphology<br>- Organismal Development |

**Table S6B: Top molecular and cellular functions and physiological processes affected by candidate genes involved in adaptation to Low and High precipitation in the driest quarter (precDQ)**

| Low precipitation in driest quarter | High precipitation in driest quarter |
|---|---|
| **Top molecular and cellular function**<br>- Gene Expression<br>- Cell Morphology<br>- Cellular Assembly and Organization<br>- Cellular Movement<br>- Cellular Function and Maintenance | **Top molecular and cellular function**<br>- Cellular Assembly and Organization<br>- Cellular Function and Maintenance<br>- Molecular Transport<br>- Protein Trafficking<br>- Cell-To-Cell Signalling and Interaction |
| **Physiological system development and function**<br>- Organismal Development<br>- Nervous System Development and Function<br>- Organ Morphology<br>- Tissue Morphology<br>- Embryonic Development | **Physiological system development and function**<br>- Embryonic Development<br>- Organismal Development<br>- Tissue Morphology<br>- Nervous System Development and Function<br>- Organ Morphology |

**Table S6C: Top molecular and cellular functions and physiological processes affected by candidate genes involved in adaptation to Low and High precipitation seasonality (precSeasonality)**

| Low precipitation seasonality | High precipitation seasonality |
|---|---|
| **Top molecular and cellular function**<br>- Molecular Transport<br>- Cell Cycle<br>- Cell-To-Cell Signalling and Interaction<br>- Cellular Assembly and Organization<br>- Cellular Development | **Top molecular and cellular function**<br>- Cell Death and Survival<br>- Gene Expression<br>- Cell Cycle<br>- Cellular Movement<br>- Cell Morphology |
| **Physiological system development and function**<br>- Connective Tissue Development and Function<br>- Embryonic Development<br>- Skeletal and Muscular System Development and Function<br>- Tissue Development | **Physiological system development and function**<br>- Cardiovascular System Development and Function<br>- Organ Morphology<br>- Organismal Development<br>- Renal and Urological System Development and Function<br>- Embryonic Development |

**Table S6D: Top molecular and cellular functions and physiological processes affected by candidate genes involved in adaptation to Low and High precipitation in the wettest quarter (precWQ)**

| Low precipitation in wettest quarter | High precipitation in wettest quarter |
|---|---|
| **Top molecular and cellular function**<br>- Amino Acid Metabolism<br>- Carbohydrate Metabolism<br>- Cell Death and Survival<br>- Cell Morphology<br>- Cell-To-Cell Signalling and Interaction | **Top molecular and cellular function**<br>- Cellular Assembly and Organization<br>- Cellular Function and Maintenance<br>- Cell Cycle<br>- DNA Replication, Recombination, and Repair<br>- Cellular Development |
| **Physiological system development and function**<br>- Embryonic Development<br>- Organismal Development<br>- Tissue Development<br>- Organ Development<br>- Respiratory System Development and Function | **Physiological system development and function**<br>- Nervous System Development and Function<br>- Tissue Morphology<br>- Cardiovascular System Development and Function<br>- Embryonic Development<br>- Organ Development |

**Table S6E: Top molecular and cellular functions and physiological processes affected by candidate genes involved in adaptation to Low and High soil organic carbon content (SoilOrgC)**

| Low soil organic carbon content | High soil organic carbon content |
|---|---|
| **Top molecular and cellular function**<br>- Cellular Function and Maintenance<br>- Molecular Transport<br>- Cellular Assembly and Organization<br>- Cell-To-Cell Signalling and Interaction<br>- Cell Death and Survival | **Top molecular and cellular function**<br>- Cell-To-Cell Signalling and Interaction<br>- Cellular Assembly and Organization<br>- Cellular Function and Maintenance<br>- Cellular Development<br>- Cellular Growth and Proliferation |
| **Physiological system development and function**<br>- Hematological System Development and Function<br>- Tissue Development<br>- Renal and Urological System Development and Function<br>- Organismal Functions<br>- Tissue Morphology | **Physiological system development and function**<br>- Nervous System Development and Function<br>- Tissue Development<br>- Cardiovascular System Development and Function<br>- Organ Morphology<br>- Organismal development |

**Table S6F: Top molecular and cellular functions and physiological processes affected by candidate genes involved in adaptation to Low and High proportion of land use for cultivation purpose (LandUse)**

| **Low proportion of land used for cultivation** | **High proportion of land used for cultivation** |
|---|---|
| **Top molecular and cellular function**<br>- Nucleic Acid Metabolism<br>- Small Molecule Biochemistry<br>- Molecular Transport<br>- Cellular Movement<br>- Drug Metabolism | **Top molecular and cellular function**<br>- Cell-To-Cell Signalling and Interaction<br>- Cellular Assembly and Organization<br>- Amino Acid Metabolism<br>- Lipid Metabolism<br>- Molecular Transport |
| **Physiological system development and function**<br>- Embryonic Development<br>- Organismal Development<br>- Nervous System Development and Function<br>- Organ Development<br>- Tissue Development | **Physiological system development and function**<br>- Behaviour<br>- Embryonic Development<br>- Nervous System Development and Function<br>- Organismal Development Tissue Development |

**Table S7: Candidate SNPs from RDA analysis**

See worksheets: “TableS7A_RDA_outlierSNPs_r0.3” and “TableS7B_SNPs-inLD-with_RDAcand” under the supplementary file “SI_Tables_S3_S4_S5_S7.xlsx”

**Table S8: Major candidate genes associated with different environmental predictors from RDA analysis. The list include those genes associated with SNPs with *r* value ≥ 0.4 with an environmental predictor or are common between at least one SSA approach and RDA analysis with environmental *r* value >=0.3 (shown with *).**

| Candidate genes | Associated SNP, type & correlation | Gene function |
|---|---|---|
| ***minTemp*** | | |
| *VMP1** | chr19_7458372 (intronic); r=0.36 | Stress induced protein, involved in autophagy (Uniprot) |
| *SEPT9** | chr18_3814601 (intronic); r=0.31 | Functions as a master transcriptional regulator of the adaptive response to hypoxia (Uniprot) |
| ***precDQ*** | | |
| *GDPD1* | chr19_7360154 (intronic); r=0.42 | Glycerophospholipid catabolic process; phospholipid metabolic process (Uniprot); in some plant species the gene is upregulated in drought stress (Kotrade, et al. 2019). |
| *HMGCLL1** | chr3_87500084; (intronic); r=0.41 | Involved in ketone body biosynthetic process (Uniprot). Ketone body - β-hydroxybutyrate, acts as a stress response molecule and plays an essential role in maintaining redox homeostasis in body under environmental and metabolic challenges (Rojas-Morales, et al. 2020) |
| *ENSGALG 00000052510* | chr2_7014078; chr2_7013782 (both ncRNA_intronic); r=0.41 | LncRNA with possible cis-regulatory role on nearby protein-coding genes. |
| *CASQ2** | chr1_81277975 (intronic); r=0.34 | Involved in regulation of skeletal muscle contraction by regulation of release of sequestered calcium ion; response to heat; regulation of cardiac conduction (Uniprot) |
| *ENSGALG 00000046751** | chr12_18108027 (ncRNA_intronic); r=0.31 | LncRNA with possible cis-regulatory role on nearby protein-coding genes. |
| ***precWQ*** | | |
| *GPC5* | chr1_149407037 (intronic); r=0.47 | Positive regulation of canonical Wnt signaling pathway (Uniprot); this pathway negatively regulates stress granule assembly in cells (7). GPC5 gene has been found associated with renal disease (Okamoto, et al. 2015) |
| *PPFIA2* | chr1_40560346 (intronic): r=0.46 | Regulation of dendritic spine development; neurotransmitter secretion (Uniprot) |
| *MRPL46* | chr10_13635900 (intergenic); r=0.41 | The encoded protein is a structural constituent of mitochondrial ribosome (GeneCards ; Uniprot) |
| *PHLPP1** | chr2_68078952 (intronic); r=0.31 | Regulation of apoptotic process (Uniprot) |
| ***precSeasonality*** | | |
| *ENSGALG 00000047134* | chr4_40753420 (intergenic); r=0.40 | LncRNA with possible cis-regulatory role on nearby protein-coding genes. |
| *ENSGALG 00000049389** | chr3_50886930 (intergenic); r=0.35 | LncRNA with possible cis-regulatory role on nearby protein-coding genes. |

| | | |
|---|---|---|
| *SLIT3** | chr13_5424064 (intronic); r=0.33 | Many functions including apoptotic process, atrioventricular valve morphogenesis, axon guidance, cellular response to hormone stimulus, negative chemotaxis, negative regulation of cell growth,<br>negative regulation of cell population proliferation, response to cortisol, Roundabout signalling pathway (1) |
| *SLK** | chr6_25081534 (intronic); r=0.33 | *SLK* - mediates apoptosis (Uniprot) |
| ***SoilOrgC*** | | |
| *KIF5C* | chr7_34788668 (intronic), *r*=0.52 | Involved in synaptic transmission, axonal protein transport, axon guidance (1) |
| *SPARC* | chr13_13644287 (intergenic); r=0.52 | Regulate cell growth through interactions with the extracellular matrix and cytokines. Involved in many biological processes including cellular response to growth factor stimulus, anatomical structure development, inner ear development, response to peptide hormone, response to lipopolysaccharide, response to glucocorticoid, bone development, regulation of synapse organization (1) |
| *ENSGALG 00000048256* | chr15_10037728 (intergenic); r=0.52 | LncRNA with possible cis-regulatory role on nearby protein-coding genes. |
| *ENSGALG 00000053295* | chr7_34393633 (ncRNA_intronic); r=0.49 | LncRNA with possible cis-regulatory role on nearby protein-coding genes. |
| *ZEB2** | chr7_33312018 (intronic); r=0.48 | Many important functions in brain and nervous system development and processes. Other roles include positive regulation of lens fiber cell differentiation, positive regulation of melanin biosynthetic process, and melanocyte differentiation (1) |
| *HMGA2* | chr1_34440130 (intergenic); r=0.46 | Functions as transcriptional regulator affecting many biological functions (1) |
| *CMSS1* | chr1_85864551 (intronic); r=0.44 | RNA binding (Uniprot) |
| *ENSGALG 00000054669* | chr1_118488638 (intergenic); r=0.44 | LncRNA with possible cis-regulatory role on nearby protein-coding genes. |
| *ENSGALG 00000048894** | chr1_171071423 (intergenic); r=0.44 | LncRNA with possible cis-regulatory role on nearby protein-coding genes. |
| *RAB5A* | chr2_35770543 (intronic); r=0.45 | Many function, including involved in early endosome to late endosome transport, regulation of endocytosis, regulation of long-term neuronal synaptic plasticity (Uniprot) |
| *ENSGALG 00000029333* | chr3_4543355 (ncRNA_intronic); r=0.44 | LncRNA with possible cis-regulatory role on nearby protein-coding genes. |
| *CCNC* | chr3_71404526 (upstream); r=0.44 | Transcription regulation (Uniprot) |
| *COQ3* | chr3_71519113 (intronic); r=0.44 | Glycerol metabolic process; methylation; regulation of ubiquinone biosynthetic process (Uniprot) |
| *FAM13A* | chr4_35187554 (intronic); r=0.44 | Signal transduction; possible role in body fat distribution (Tang, et al. 2019) |

| | | |
|---|---|---|
| *TENM3** | chr4_40119898 (intronic); r=0.44 | Neural development, camera-type eye morphogenesis (1) |
| *EPB41* | chr23_2798748 (intronic); r=0.44 | The encoded protein is an important structural element of erythrocyte membrane skeleton (1). Invovled in regulation of intestinal absorption (1) and a candidate for feed coversion efficiency in pigs (Horodyska, et al. 2017) |
| *ENSGALG 00000052236* | chr24_773894 (downstream); r=0.44 | LncRNA with possible cis-regulatory role on nearby protein-coding genes. |
| *NUMA1* | chr1_195605868 (downstream); r=0.43 | Microtubule binding protein playing important role during mitotic cell division and chromosome segregation. |
| *ENSGALG 00000036131* | chr1_52325047 (intergenic); r=0.42 | LncRNA with possible cis-regulatory role on nearby protein-coding genes. |
| *GABRB3* | chr1_132840375 (intergenic); r=0.42 | Involved in nervous system process (1) |
| *PEX1* | chr2_22649717 (UTR3); r=0.42 | Plays important role in peroxisome biogenesis (GeneCards); peroxisomes play vital roles in a variety of metabolic pathways such as lipid and amino acid metabolisms, and reactive oxygen species metabolism (Wanders and Waterham 2006) |
| *CDH6* | chr2_69280226 (intronic); r=0.42 | Encodes a cadherin protein; Cadherins are membrane glycoproteins that mediate homophilic cell-cell adhesion and play critical roles in cell differentiation and morphogenesis (GeneCards). |
| *ADGRA3* | chr4_74483560 (intronic); r=0.42 | Encodes a G protein-coupled receptor (GPCR) (Uniprot); GPCRs mediate senses such as vision, smell, taste, and pain and are also involved in cell recognition and communication processes (Vaidehi, et al. 2002) |
| *ENSGALG 00000007683* | chr4_12164664 (intronic); r=0.42 | Novel protein coding gene |
| *RABGAP1L* | chr8_7489597 (intergenic); r=0.42 | Endocytosis, intracellular protein transport, regulation of protein localization (1) |
| *ITGA11* | chr10_19610334 (intronic); r=0.42 | Encoded protein is a collagen receptor, with functions in muscle organ development and osteoblast differentiation (1) |
| *KIF23* | chr10_19801266 (intergenic); r=0.42 | Involved in mitotic cytokinesis - a cell cycle process that results in the division of the cytoplasm of a cell (1) |
| *PAPPA* | chr17_3435838 (intronic); r=0.42 | Cellular protein metabolic process, response to follicle-stimulating hormone; response to glucocorticoid (1) |
| *MDGA1* | chr3_30160547 (intronic); r=0.41 | Brain development, neuron migration, spinal cord association neuron differentiation (1) |
| *ENSGALG 00000050118* | chr9_5405891 (ncRNA_intronic); r=0.41 | LncRNA with possible cis-regulatory role on nearby protein-coding genes. |
| *ENSGALG 00000050572* | chr17_4243280 (ncRNA_intronic); r=0.41 | LncRNA with possible cis-regulatory role on nearby protein-coding genes. |
| *ACVR2B** | chr2_5890638 (intronic); r=0.40 | Involved in many biological processes associated with growth and development e.g. neuronal |

| | | |
|---|---|---|
| | | development, cellular response to growth factor stimulus, embryonic foregut morphogenesis, insulin secretion, various organs development, pancreas development, positive regulation of bone mineralization, response to glucose (1). |
| *CDYL* | chr2_65612774 (intronic): r=0.40 | Chromatin binding, transcription corepressor activity, spermatogenesis (1) |
| *ENSGALG 00000054427* | chr8_27387228 (intergenic); r=0.40 | LncRNA with possible cis-regulatory role on nearby protein-coding genes. |
| *ENSGALG 00000054932* | chr20_4834422 (downstream); r=0.40 | LncRNA with possible cis-regulatory role on nearby protein-coding genes. |
| *ETV3* | chr25_2525881 (intronic); r=0.40 | Transcriptional regulation, negative regulation of cell population proliferation (1) |
| *PABPC1** | chr2_128516548 (intergenic); r=0.37 | Translational initiation; gene silencing by RNA (1) |
| *CACNB4** | chr7_35357016 (intronic); r=0.36 | Regulation of voltage-gated calcium channel activity, with roles in cardiac conduction, chemical synaptic transmission, neuromuscular junction development (1) |
| *ARPC1A** | chr14_4758137 (intronic); r=0.36 | Actin cytoskeleton organization, ephrin receptor signaling pathway (1) |
| *ENSGALG 00000050593** | chr6_25343111 (ncRNA_Intronic); r=0.35 | |
| *FARP1** | chr1_146510496 (intronic); r=0.32 | Dendrite morphogenesis, synapse assembly (1) |
| ***LandUse*** | | |
| *GDPD4* | chr1_194559771 (upstream); r=0.40 | Involved in lipid metabolic process (1) |
| *ITGB1* | chr2_14079356 (intronic); r=0.40 | Part of Integrin family of proteins, which are membrane receptors involved in cell adhesion and recognition in a variety of processes including nervous system processes, embryogenesis, hemostasis, tissue repair, immune response, and visual learning (1). |
| *GABRG3** | chr1_132488743, (intronic SNPs): r=0.33 | Involved in nervous system process (1) |
| *STAU2** | chr2_117937265 (intronic); r=0.31 | RNA binding associated with neuronal activity (1) |

## Supplementary References


Balloux F, Lugon-Moulin N. 2002. The estimation of population differentiation with microsatellite markers. *Mol. Ecol.* 11:155-165.

Browning SR, Browning BL. 2007. Rapid and accurate haplotype phasing and missing-data inference for whole-genome association studies by use of localized haplotype clustering. *Am. J. Hum. Genet.* 81:1084-1097.

Elferink MG, van As P, Veenendaal T, Crooijmans RPMA, Groenen MAM. 2010. Regional differences in recombination hotspots between two chicken populations. *BMC Genet.* 11.

Fick SE, Hijmans RJ. 2017. WorldClim 2: new 1-km spatial resolution climate surfaces for global land areas. *Int J Climatol.* 37:4302-4315.

Fischer G, Nachtergaele FO, Prieler S, Teixeira E, Tóth G, van Velthuizen H, Verelst L, Wiberg D. 2008. Global Agro-ecological Zones Assessment for Agriculture (GAEZ 2008). IIASA, Laxenburg, Austria and FAO, Rome, Italy.

Forester BR. 2019. Detecting multilocus adaptation using Redundancy Analysis (RDA); https://popgen.nescent.org/2018-03-27_RDA_GEA.html; Accessed on 24/11/2019.

GeneCards. https://www.genecards.org/: accessed on 29/10/2020.

GFSAD30. 2017. Global Food Security Analysis-Support Data at 30 Meters Project. https://www.usgs.gov/centers/wgsc/science/global-food-security-support-analysis-data-30-m/ Accessed 21/11/2019.

Groenen MAM, Wahlberg P, Foglio M, Cheng HH, Megens HJ, Crooijmans RPMA, Besnier F, Lathrop M, Muir WM, Wong GKS, et al. 2009. A high-density SNP-based linkage map of the chicken genome reveals sequence features correlated with recombination rate. *Genome Res.* 19:510-519.

Hartl DL, Clark AG. 1997. Principles of Population Genetics, 3rd edn. Sinauer Associates, Inc, Sunderland, MA.

Hengl T, de Jesus JM, MacMillan RA, Batjes NH, Heuvelink GBM, Ribeiro E, Samuel-Rosa A, Kempen B, Leenaars JGB, Walsh MG, et al. 2014. SoilGrids1km-Global Soil Information Based on Automated Mapping. *PLoS One* 9 (12): e114788..

Horodyska J, Hamill RM, Varley PF, Reyer H, Wimmers K. 2017. Genome-wide association analysis and functional annotation of positional candidate genes for feed conversion efficiency and growth rate in pigs. *PLoS One* 12(6): e0173482.

Jueterbock A, Smolina I, Coyer JA, Hoarau G. 2016. The fate of the Arctic seaweed Fucus distichus under climate change: an ecological niche modeling approach. *Ecol Evol.* 6:1712-1724.

Kotrade P, Sehr EM, Wischnitzki E, Bruggemann W. 2019. Comparative transcriptomics-based selection of suitable reference genes for normalization of RT-qPCR experiments in drought-stressed leaves of three European Quercus species. *Tree Genet Genomes.* 15:38.

Maclean CA, Hong NPC, Prendergast JGD. 2015. hapbin: An Efficient Program for Performing Haplotype-Based Scans for Positive Selection in Large Genomic Datasets. *Mol Biol Evol.* 32:3027-3029.

Muscarella R, Galante PJ, Soley-Guardia M, Boria RA, Kass JM, Uriarte M, Anderson RP. 2014. ENMeval: An R package for conducting spatially independent evaluations and estimating optimal model complexity for MAXENT ecological niche models. *Methods Ecol. Evol.* 5:1198-1205.

Okamoto K, Honda K, Doi K, Ishizu T, Katagiri D, Wada T, Tomita K, Ohtake T, Kaneko T, Kobayashi S, et al. 2015. Glypican-5 Increases Susceptibility to Nephrotic Damage in Diabetic Kidney. *Am. J. Pathol.* 185:1889-1898.

Oksanen J. 2015. Multivariate analysis of ecological communities in R: vegan tutorial; https://cran.r-project.org/web/packages/vegan/vegan.pdf: Accessed on 24/11/2019.

ORNL_DAAC. 2017. Spatial Data Access Tool (SDAT). ORNL DAAC, Oak Ridge, Tennessee, USA. https://doi.org/10.3334/ORNLDAAC/1388, Accessed on 24/11/2019.

Rojas-Morales P, Pedraza-Chaverri J, Tapia E. 2020. Ketone bodies, stress response, and redox homeostasis. *Redox Biol.* 29.

Tang JZ, Zhou HY, Sahay K, Xu WQ, Yang J, Zhang W, Chen WQ. 2019. Obesity-associated family with sequence similarity 13, member A (FAM13A) is dispensable for adipose development and insulin sensitivity. *Int J Obes.* 43:1269-1280.

Uniprot. https://www.uniprot.org/; accessed on 10/02/2020.

Vaidehi N, Floriano WB, Trabanino R, Hall SE, Freddolino P, Choi EJ, Zamanakos G, Goddard WA. 2002. Prediction of structure and function of G protein-coupled receptors. *PNAS.* 99:12622-12627.

Wanders RJA, Waterham HR. 2006. Biochemistry of mammalian peroxisomes revisited. *Annu Rev Biochem.* 75:295-332.
